# An EGFR/Src-dependent β4 integrin/FAK complex contributes to malignancy of breast cancer

**DOI:** 10.1038/srep16408

**Published:** 2015-11-09

**Authors:** Yu-Ling Tai, Pei-Yu Chu, I-Rue Lai, Ming-Yang Wang, Hui-Yuan Tseng, Jun-Lin Guan, Jun-Yang Liou, Tang-Long Shen

**Affiliations:** 1 Department of Plant Pathology and Microbiology, National Taiwan University, Taipei 10617, Taiwan; 2 Department of Anatomy and Cell Biology, College of Medicine, National Taiwan University, Taipei 10051, Taiwan; 3 Department of Surgery, National Taiwan University Hospital, Taipei 10048, Taiwan; 4 Department of Cancer Biology, University of Cincinnati College of Medicine, Cincinnati, OH 45267, USA; 5 Institute of Cellular and System Medicine, National Health Research Institutes, Zhunan, Miaoli County 35053, Taiwan; 6 Center for Biotechnology, National Taiwan University, Taipei 10617, Taiwan

## Abstract

β4 integrin and focal adhesion kinase (FAK) are often associated with a poor prognosis in cancer patients, and their signaling events have recently been linked to malignant outcomes. Here, we demonstrate, for the first time, physical and functional interactions between β4 integrin and FAK that influence breast cancer malignancy. An amino-terminal linker within FAK is essential for its binding with the cytodomain of β4 integrin. Moreover, EGFR/Src-signaling triggers the tyrosine phosphorylation of β4 integrin, which, in turn, recruits FAK to β4 integrin and leads to FAK activation and signaling. Upon disruption of the β4 integrin/FAK complex, tumorigenesis and metastasis in triple-negative breast cancer were markedly reduced. Importantly, the concomitant overexpression of β4 integrin and FAK significantly correlates with malignant potential in patients with triple-negative breast cancer. This study describes a pro-metastatic EGFR/Src-dependent β4 integrin/FAK complex that is involved in breast cancer malignancy and is a novel therapeutic target for triple-negative breast cancer.

Breast cancer is a progressive and heterogeneous disease worldwide. Based on molecular analyses and clinical outcomes, this disease is classified into five distinct subtypes: luminal A (estrogen receptor positive, ER^+^ and/or progesterone receptor positive, PR^+^, and human epidermal growth factor receptor-2 negative, HER2^−^), luminal B (ER^+^ and/or PR^+^, and HER2^+^), HER2 over-expressing (ER^−^ and/or PR^−^, and HER2^+^), triple-negative (ER^−^, PR^−^, and HER2^−^), and unclassified^1^. Of these, mainly triple-negative breast cancer (TNBC) is associated with aggressive malignancy, high rates of recurrence, and the worst prognosis^2^. However, there are currently few effective therapeutics for patients with TNBC due to a lack of proper targets for treatment strategies. Therefore, it is critical to reveal the underlying mechanisms that confer malignancy in TNBCs, which will facilitate the development of potent anti-cancer therapeutics.

The β4 integrin subunit associates with α6 integrin to act on the assembly of hemidesmosomes in epithelial cells^3^. Nevertheless, β4 integrin was initially identified as a tumor-related antigen that is expressed in metastatic cancer^4^ and is correlated with malignant progression in cancers, including breast cancer, colorectal cancer, and lung cancer^5^,^6^,^7^. Recent studies indicate that β4 integrin significantly correlates with the development and prognostic significance of TNBC^8^. Indeed, the cytoplasmic domain of β4 integrin is known to bind with Shc, and Shp2 contributes to the activation of MAPK cascades to promote tumor malignancy^9^,^10^. Subsequent functional studies provide mechanistic support for β4 integrin-mediated Ras and MAPKs activation, which modulates breast cancer proliferation and invasion^11^,^12^. Moreover, β4 integrin signaling may drive breast carcinoma resistance to apoptosis-inducing and anti-HER2 agents^13^,^14^, implying that β4 integrin signaling is important in the development of breast cancer malignancy.

FAK is a non-receptor tyrosine kinase that is critical for integrin-mediated signaling and cellular functions. FAK also functions as a convergent point for various signaling pathways that are associated with cell adhesion, migration, and oncogenic transformation^15^,^16^,^17^. It is generally reported that overexpression and auto-phosphorylation of FAK are involved in the development of malignancy in various cancers^18^,^19^. In particular, a recent study has indicated that FAK overexpression is significantly associated with high histologic grade, especially in the triple-negative subtype of breast cancer^20^, which is consistent with reports that the *Fak* gene is often up-regulated in TNBC^21^. In agreement with a role for FAK in tumor progression, several studies have attempted to block FAK activity to inhibit various FAK-mediated tumor malignancies^22^,^23^, to explore FAK as a novel target for anti-cancer therapy.

Accumulating evidence indicates that overexpression of β4 integrin or FAK is intimately associated with the malignancy of breast cancer^11^,^24^. Recent studies by Abdel-Ghany *et al.* revealed that β4 integrin enables the modulation of FAK-mediated signaling during the regulation of β4 integrin-dependent tumorigenesis and malignancy^25^. Nevertheless, the clinical relevance and the molecular mechanism of the association of β4 integrin and FAK that contributes to the malignancy of TNBC remains elusive. In this study, we illustrate a molecular signaling cascade in which the EGF-Src-β4 integrin axis physically recruits and activates FAK activity and downstream signaling, thereby facilitating the progression of breast cancer towards malignancies.

## Results

### The physical interaction between β4 integrin and FAK correlates with tumor malignancy

**Figure 1:**
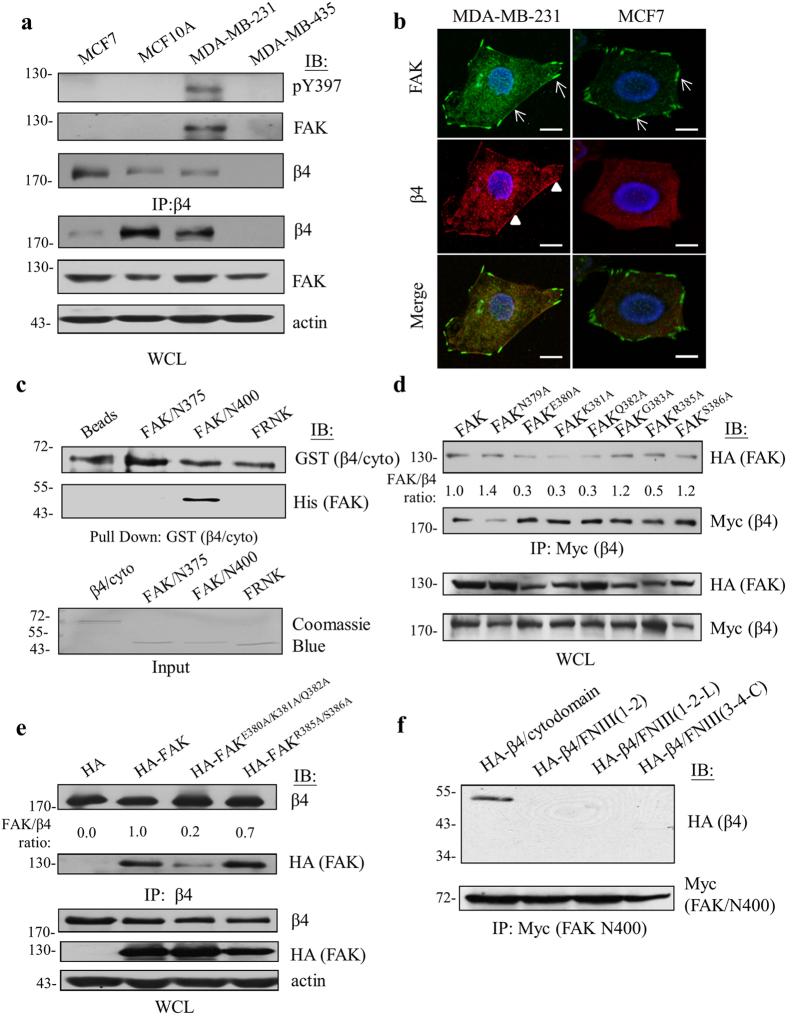
The physical interaction of β4 integrin and FAK is associated with tumor malignancy *in vivo* and *in vitro.* (**a**) Varied human cancer cell lines were analyzed by Western blot analysis with anti-β4 integrin, anti-FAK, or anti-phospho-Tyr397 antibody, showing an interaction between β4 integrin and FAK. The human mammary epithelial cell line MCF10A was used as a normal control. (**b**) MDA-MB-231 (aggressive) and MCF7 (non-aggressive) cells were stained to show the co-localization of FAK (green, arrows) and β4 integrin (red, arrowheads) on the peripheral plasma membrane in MDA-MD-231 cells but not in MCF7 cells. Scale bars, 10 μm. (**c**) The association between β4 integrin and FAK-derived recombinant proteins was determined by an *in vitro* binding assay. (**d**) By immunoprecipitation and Western blot analysis, the crucial amino acids that were responsible for interaction with β4 integrin were determined. The mean of the relative interaction between β4 integrin and FAK (normalized to wild-type FAK shown as 1.0) was measured. (**e**) The triple amino acids (FAK^E380A/K381A/Q382A^) exhibited a marked reduction in β4 integrin binding compared to wild-type FAK or the double (FAK^R385A/S386A^) mutant. (**f**) The cytodomain of β4 integrin is indispensable to its interaction with FAK. Each experiment was repeated at least three independent times and the densitometric analysis of the relative quantification of band intensities, normalized to respective controls, is shown in Supplementary Fig. S6. All cropped blots were run under the same experimental conditions. The full-length blots are included in Supplementary Fig. S7.

The putative interaction between β4 integrin and FAK in relation to tumor malignancy was analyzed by immunoprecipitation in varied cancer cell lines. The interaction between β4 integrin and FAK was identified in the malignant triple-negative breast cancer cell line (MDA-MB-231) (Fig. 1a) as well as in the metastatic colon cancer cell line (HCT-116) (unpublished data), but not in the non-tumorigenic breast epithelial cell line (MCF10A) or other cancer cell lines (i.e., MCF7, MDA-MB-435, A549, and HeLa). In addition, we also observed that β4 integrin and FAK were co-localized in the plasma membrane or protrusions of metastatic breast MDA-MB-231 cells in contrast to that observed in the non-metastatic breast MCF7 cells (Fig. 1b). Together, these results indicate that β4 integrin might interact with FAK in MDA-MB-231 breast cancer cells.

To clarify the physical interaction between β4 integrin and FAK, we demonstrated that the recombinant His-tagged FAK/N400 was capable of precipitating β4 integrin from MDA-MB-231 cells, but neither His-tagged FAK/N375 nor His-tagged FRNK as precipitated (Supplementary Fig. S1a). Using a far-Western assay and an *in vitro* pull-down assay, we further supported a direct interaction between β4 integrin and FAK that required a sequence within the first 400 amino acids of FAK, but not within the first 375 amino acids, and the cytodomain of β4 integrin (Fig. 1c and Supplementary Fig. S1b). Together, these results support, for the first time, a physical link between the cytodomain of β4 integrin and a 25-amino-acid motif within FAK’s N-terminus that is present in triple-negative breast cancers.

In light of the above findings, we further dissected the binding sites for these two molecules. First, we generated serial FAK truncated mutants and subjected them to co-immunoprecipitation assays with β4 integrin. Collectively, as summarized in Supplementary Fig. S1c, we found that an 11-amino-acid region (*i.e.*, from the 376^th^ to the 386^th^ amino acid) ahead of the FAK-Tyr^397^ autophosphorylation site is responsible for β4 integrin binding (Supplementary Fig. S1d–f). Furthermore, we mapped the essential amino acids of the 11 amino acids as Leu^376^-Ala-Asn-Asn-Glu-Lys-Gln-Gly-Val-Arg-Ser^386^, and showed that they are critically involved in the interaction with β4 integrin by using a site-directed mutagenesis approach to convert individual amino acids into alanine. As a result, three (Glu^380Ala^, Lys^381Ala^, and Gln^382Ala^) out of 10 FAK alanine mutants significantly diminished its ability to bind with β4 integrin in comparison to wild-type FAK (Fig. 1d). Then, we generated and tested the β4 integrin binding ability of the FAK triple-point-mutation mutant, FAK^E380A/K381A/Q382A^, and the control double-point-mutation mutant, FAK^R385A/S386A^, which consistently supported an essential role for the Glu^380^, Lys^381^, and Gln^382^ residues for interacting with β4 integrin (Fig. 1e).

We searched for the FAK binding site on β4 integrin by co-immunoprecipitation using distinctive regions derived from the β4 integrin cytoplasmic domain, such as the cytodomain, the FNIII (1–2), the FNIII (1–2-L), and the FNIII (3–4-C). As summarized in Supplementary Fig. S1h, we determined that the FAK binding site of β4 integrin resided in the cytodomain of β4 integrin, proximal to the plasma membrane rather than to the FNIII repeats or the linker region (Fig. 1f and Supplementary Fig. S1g).

### EGF/Src signaling regulates β4 integrin phosphorylation and β4 integrin/FAK complex formation

**Figure 2:**
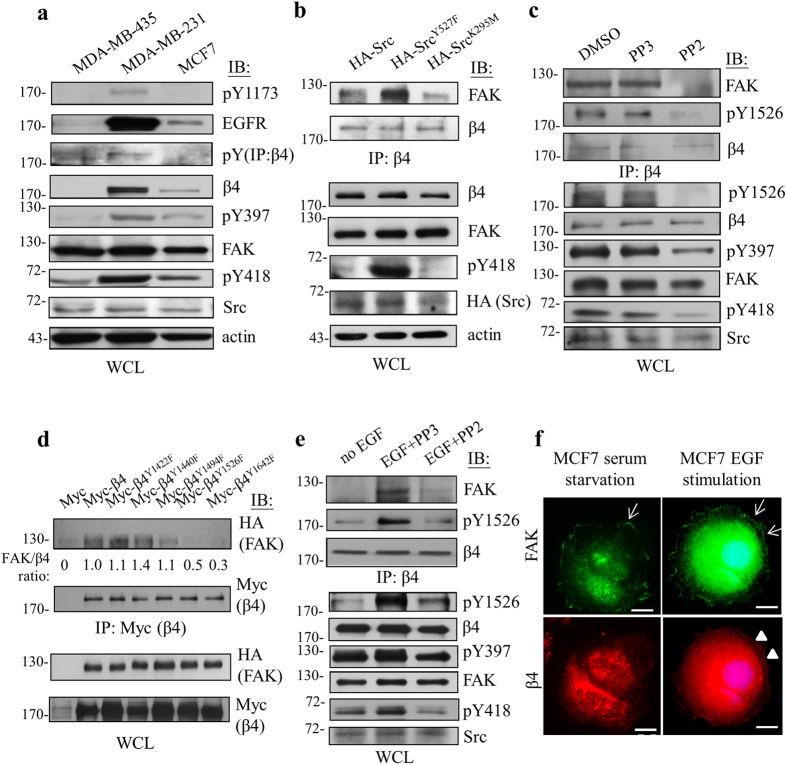
EGF/Src-dependent β4 integrin phosphorylation modulates the formation of the β4 integrin/FAK complex. (**a**) Varied human cancer cell lines were subjected to Western blot analysis to analyze the phosphorylation and expression of indicated signal molecules. (**b**) MDA-MB-231 cells were transfected with HA-tagged wild-type Src, constitutively active Src^Y527F^, and kinase-dead Src^K295M^ to examine the effect of Src kinase activity on the interaction between β4 integrin and FAK. The results indicated that Src kinase activity promotes the interaction between β4 integrin and FAK. (**c**) MDA-MB-231 cells were treated with DMSO, PP2 (10 μM), or PP3 (10 μM) to examine the effect of Src kinase activity on the tyrosine phosphorylation of β4 integrin and FAK as well as the interaction between β4 integrin and FAK. (**d**) Phospho-tyrosine point mutation mutants of β4 integrin, as indicated, were analyzed to examine their competence for interacting with FAK. The mean of relative interaction between β4 integrin and FAK (normalized to wild-type β4 integrin shown as 1.0) was measured. (**e**) Serum-starved MDA-MB-231 cells were stimulated with EGF (10 ng/ml) in the presence of PP2 (10 μM) or PP3 (10 μM) to examine the effect of EGF/Src signaling on the tyrosine phosphorylation of β4 integrin and FAK as well as the interaction between β4 integrin and FAK. (**f**) Serum-starved MCF7 cells were treated with EGF (10 ng/ml) to examine the co-localization of β4 integrin (red) and FAK (green) by immunofluorescent staining. Arrows indicate the distribution of FAK at focal adhesions and/or on the peripheral plasma membrane. Arrowheads indicate the localization of β4 integrin on the plasma membrane. Scale bars, 20 μm. Each experiment was repeated at least three independent times. The densitometric analysis of the relative quantification of band intensities, normalized to respective controls, is shown in Supplementary Fig. S6. All cropped blots were run under the same experimental conditions. The full-length blots are included in Supplementary Fig. S7.

The discrepancy between metastatic MDA-MB-231 cells and non-metastatic MCF7 cells in the β4 integrin/FAK complex formation prompted us to investigate the regulatory mechanisms involved in the formation of this complex. Consistent with previous studies^25^,^26^,^27^, we observed that EGFR autophosphorylation at Tyr^1173^, Src phosphorylation at Tyr^418^, tyrosine phosphorylation of β4 integrin, and FAK autophosphorylation at Tyr^397^ were more prevalent in MDA-MB-231 cells compared to MCF7 cells (Figs 1a and 2a), implying that a tyrosine phosphorylation cascade through EGF/Src-family kinases (SFKs) is associated with β4 integrin/FAK complex formation. To explore this possibility, we directly tested the role of Src kinase activity on β4 integrin/FAK complex formation. First, we found that the interaction between β4 integrin and FAK was elevated in the presence of constitutively active Src^Y527F^ compared to the wild-type or kinase dead (Src^K295M^) Src-expressing MDA-MB-231 cells (Fig. 2b). In accordance with this finding, the formation of the β4 integrin/FAK complex was markedly reduced in the presence of PP2, an Src kinase inhibitor, compared to PP3- or DMSO-treated cells (Fig. 2c). Moreover, the phosphorylation levels of β4 integrin at Tyr^1526^ and FAK at Tyr^397^ were also reduced upon blocking Src kinase activity. Indeed, we revealed that two (Tyr^1526^ and Tyr^1642^) out of five potential SFK-mediated tyrosine phosphorylation sites in the β4 integrin signaling domain were intimately associated with the formation of the β4 integrin/FAK complex (Fig. 2d). Collectively, these results show that Src kinase activity is involved in the interaction between β4 integrin and FAK.

Tyrosine phosphorylation of β4 integrin by SFKs is reportedly important for the progression of tumor malignancy in a growth factor-dependent manner^14^,^26^. We next attempted to determine whether EGF/EGFR signaling enables the control of Src-mediated the β4 integrin/FAK complex formation. As shown in Fig. 2e, the interaction between β4 integrin and FAK was virtually increased by EGF stimulation but remained absent in the presence of PP2, indicating that EGF/Src-mediated signaling was involved in the β4 integrin/FAK complex formation. In accordance with the tyrosine phosphorylation regulatory cascade, we also observed that the tyrosine phosphorylation of β4 integrin and FAK was also affected by EGF/Src-mediated signaling. Moreover, upon EGF stimulation, the concomitant increases in Src phospho-Tyr^418^, β4 integrin phospho-Tyr^1526^ and FAK phospho-Tyr^397^ (Supplementary Fig. S2a) coincided with the co-localization in the plasma membrane of β4 integrin and FAK in MCF7 cells (Fig. 2f). Moreover, the same phenomenon was investigated in EGF-stimulated MDA-MB-231 cells, which showed the same co-localization at plasma membrane of β4 integrin and FAK in (Supplementary Fig. S2b). Taken together, our findings revealed that an intrinsic phospho-tyrosine cascade that is triggered by an EGF/Src-mediated signaling enables transduction through the β4 integrin/FAK complex.

### Interaction with β4 integrin enhances the activity and downstream signaling of FAK

**Figure 3:**
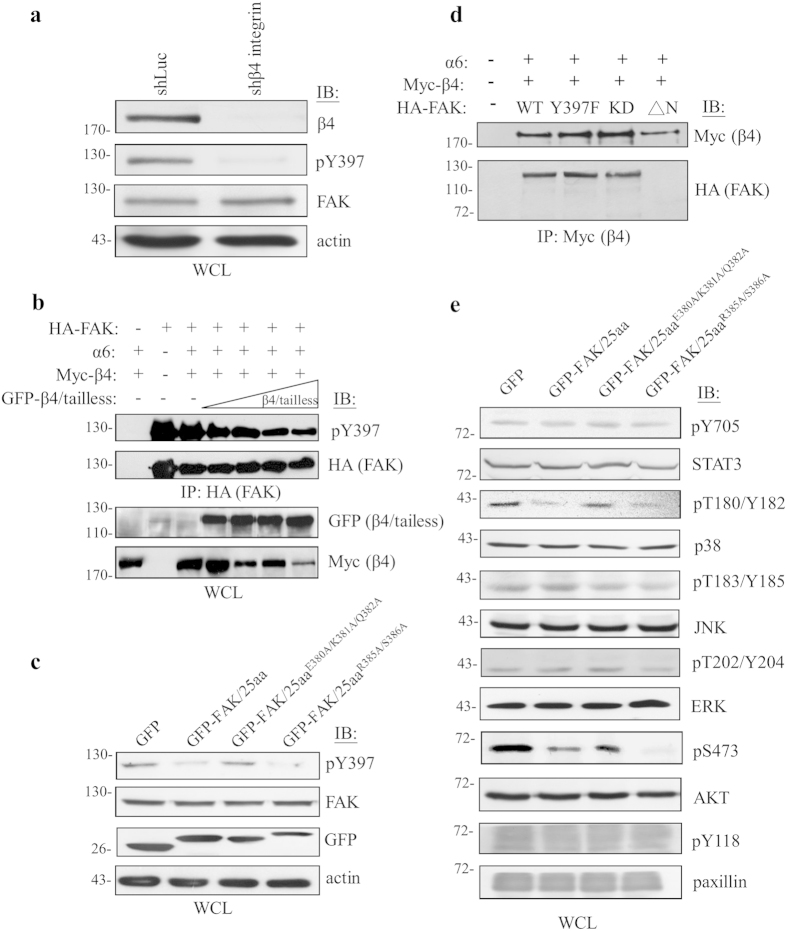
Interaction with β4 integrin leads to the activation of FAK and its downstream signaling. (**a**) Cell lysates from shLuc- or shβ4 integrin-infected MDA-MB-231 cells were subjected to Western blot analysis with anti-β4 integrin, anti-FAK, and anti-phospho-Tyr397 antibodies to examine the effect on the tyrosine phosphorylation of FAK. (**b**) The phospho-Tyr^397^ level was decreased in proportion with the increase of β4 integrin/tailless expression. (**c**) FAK/25aa (the 376^th^ to the 400^th^ amino acids), or its triple (FAK/25aa^E380A/K381A/Q382A^) or double (FAK/25aa^R385A/S386A^) mutant effects on full-length FAK phosphorylation are shown. (**d**) FAK kinase activity and phospho-Tyr^397^ were not a prerequisite for interacting with β4 integrin. The ΔN, which lacks the β4 integrin binding motif, was used as a negative control. (**e**) MDA-MB-231 cells were transfected with GFP-tagged FAK/25aa, or its triple (FAK/25aa^E380A/K381A/Q382A^) or double (FAK/25aa^R385A/S386A^) mutant to reveal potential downstream signaling, including pTyr705-STAT3, pThr180/Tyr182-p38MAPK, pThr183/Tyr185-JNKMAPK, pThr202/Tyr204-ERKMAPK, pSer473-AKT, and pTyr118-paxillin. Each experiment was repeated at least three independent times and the densitometric analysis of the relative quantification of band intensities, normalized to respective controls, are shown in the Supplementary Fig. S6. All cropped blots were run under the same experimental conditions. The full-length blots are included in Supplementary Fig. S7.

**Figure 4:**
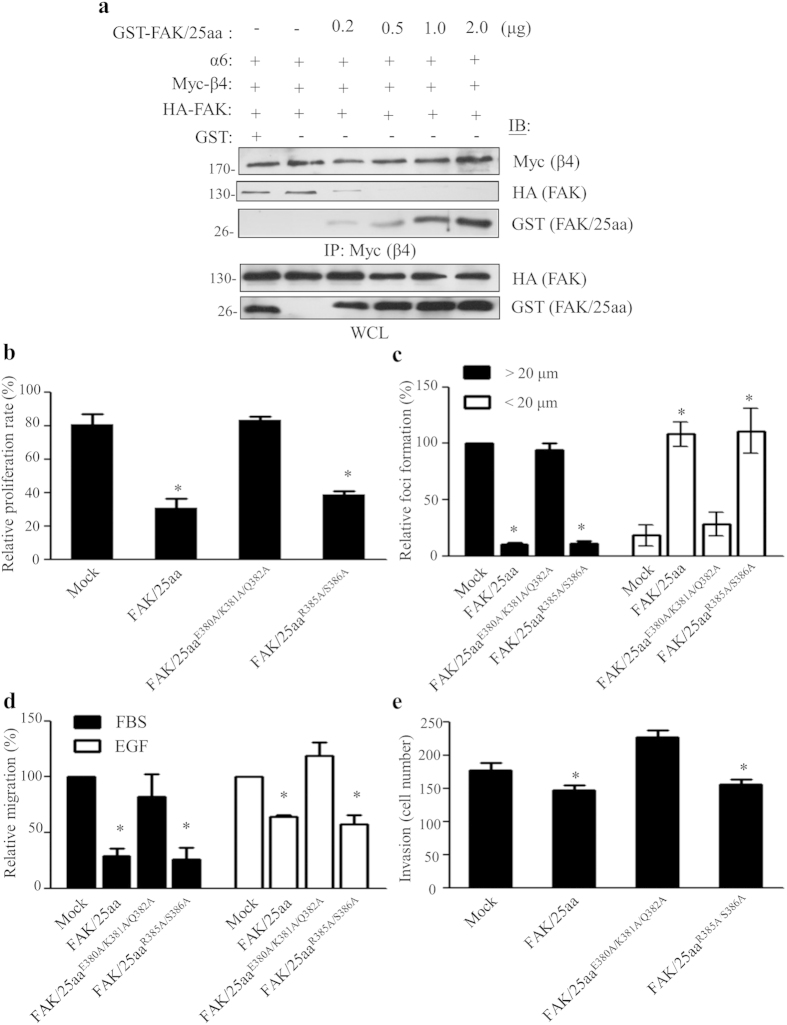
The β4 integrin/FAK complex regulates tumor malignancy *in vitro.* (**a**) The FAK/25aa peptide competes with full-length FAK for binding to β4 integrin. The experiment was repeated at least three independent times. The densitometric analysis of the relative quantification of band intensities, normalized to respective controls, is shown in Supplementary Fig. S6. All cropped blots were run under the same experimental conditions. The full-length blots are included in Supplementary Fig. S7. MDA-MB-231 cells over-expressing GFP-tagged FAK/25aa or its triple (FAK/25aa^E380A/K381A/Q382A^) or double (FAK/25aa^R385A/S386A^) mutant were subjected to cell proliferation assays using BrdU incorporation analysis, as described in Methods (**b**). A soft agar assay in the presence of EGF (10 ng/ml) was used to examine the capability for anchorage-independent growth (**c**). A cell migration assay in a modified Boyden chamber (**d**) and a Matrigel invasion assay were used to examine the capability for invasiveness in these tumor cells (**e**). All result shown as the mean ± s.d. from three independent experiments. *p < 0.05, value was in comparison to the corresponding mock control.

The downstream effects of the β4 integrin/FAK complex on breast cancer malignancy were then investigated. We noticed that the β4 integrin that co-immunoprecipitated with FAK was predominately phosphorylated at Tyr^397^ (Fig. 1a). Hence, we speculated a novel activation mechanism for FAK that included an interaction with β4 integrin via the linker motif ahead of the Try^397^ of FAK. Consistent with this, in β4 integrin knockdown cells that were deprived of the phosphorylation of FAK (Fig. 3a), the level of FAK phospho-Tyr^397^ decreased in accordance with increasing β4 integrin/tailless mutant, which can compete with full-length β4 integrin to interact with α6 integrin (Fig. 3b and Supplementary Fig. S3), due to the loss of FAK bound to β4 integrin (Supplementary Fig. S3). To further support this observation, we attempted overexpressing the FAK/25aa peptide (the 376th to the 400th amino acid), the motif for FAK that binds to β4 integrin (Fig. 1), to compete and disrupt β4 integrin/FAK complex formation in a dose-dependent manner (Fig. 4a). Meanwhile, the triple (FAK/25aa^E380A/K381A/Q382A^) and double (FAK/25aa^R385A/S386A^) FAK/25aa peptide mutants were used as controls. As expected, we found that FAK phospho-Tyr^397^ was decreased in FAK/25aa- and FAK/25aa^R385A/S386A^-transfected cells in comparison to mock or FAK/25aa^E380A/K381A/Q382A^-transfectants (Fig. 3c). In accordance, the β4 integrin/FAK complex formation was indispensable to FAK activation due to the fact that the FAK^Y397F^ or kinase dead (FAK^K454M^) mutant of FAK retained the ability to bind with β4 integrin (Fig. 3d).

Next, several potential downstream signaling targets were examined to test whether any of them participates in β4 integrin/FAK complex-mediated cancer functions, in according to previous reports^15^,^28^,^29^. As a result, AKT and p38MAPK were revealed to participate in β4 integrin/FAK complex-mediated signal transduction in triple-negative breast cancer (Fig. 3e). Collectively, these results suggest that β4 integrin enables the physical recruitment and subsequent activation of FAK, which promotes AKT and p38MAPK signaling in an EGF/Src dependent manner, thereby regulating breast cancer malignancy.

### The β4 integrin/FAK complex leads to tumor malignancy *in vitro*

The involvement of β4 integrin or FAK in tumor malignancy is well documented^14^,^15^,^30^,^31^. Thus, along with our findings, overexpression of FAK/25aa, which decreases β4 integrin/FAK complex formation and FAK activation (Fig. 3), allowed us to examine the biological effects of the β4 integrin/FAK complex. As expected, the level of FAK co-immunoprecipitated by β4 integrin was attenuated in a dose-dependent manner that correlated with increased expression of FAK/25aa (Fig. 4a). Conversely, the amounts of FAK/25aa co-immunoprecipitated by β4 integrin were increased, indicating that the reduction of the β4 integrin/FAK complex is a result of FAK/25aa competing with full-length FAK to bind with β4 integrin. It should be noted that the specificity of FAK/25aa in influencing the β4 integrin/FAK complex was affirmed because the paxillin/FAK complex and β1 integrin-mediated cell migration were not affected when FAK/25aa was overexpressed in NIH3T3 cells (Supplementary Fig. S4a).

Then, we performed various functional assays to evaluate the role of the β4 integrin/FAK complex in tumor malignancy in MDA-MB-231 cells. We found that cell proliferation was decreased in FAK/25aa and the FAK/25aa^R385A/S386A^ transfectants compared to mock and the FAK/25aa^E380A/K381A/Q382A^-transfected cells (Fig. 4b), implicating the β4 integrin/FAK complex in promoting cancer cell proliferation. In addition, the β4 integrin/FAK complex had a profound impact on enhancing the anchorage-independent growth of MDA-MB-231 cells (Fig. 4c). Concurrent with the critical role of FAK in cell migration, the β4 integrin/FAK complex had a progressive effect on cell migration toward fetal bovine serum (FBS) or EGF (Fig. 4d and Supplementary Fig. S4b). In agreement with the above, the β4 integrin/FAK complex clearly participated in tumor invasion in MDA-MB-231 cells (Fig. 4e). Collectively, the β4 integrin/FAK complex serves is a crucial candidate for identifying breast cancer malignancies.

Given that the β4 integrin/FAK complex enables the activation of AKT- and p38MAPK-mediated signaling (Fig. 3e), the distinct role of AKT and p38MAPK in β4 integrin/FAK complex-mediated cancer malignancy was investigated. By using pharmacological inhibitors, i.e., an AKT inhibitor (AKT-in) and a p38MAPK inhibitor (SB203580), we found that AKT, but not p38MAPK, is involved in β4 integrin/FAK complex-mediated anchorage-independent growth under EGF-stimulated conditions (Supplementary Fig. S4c). On the other hand, p38MAPK was required for cell migration toward EGF (Supplementary Fig. S4d). Taken together, our data reveals the molecular mechanism through which an intrinsic tyrosine phosphorylation cascade of the EGF/Src-mediated β4 integrin/FAK complex is involved in the development of breast cancer malignancy.

### The β4 integrin/FAK complex is involved in tumor malignancy *in vivo*

**Figure 5:**
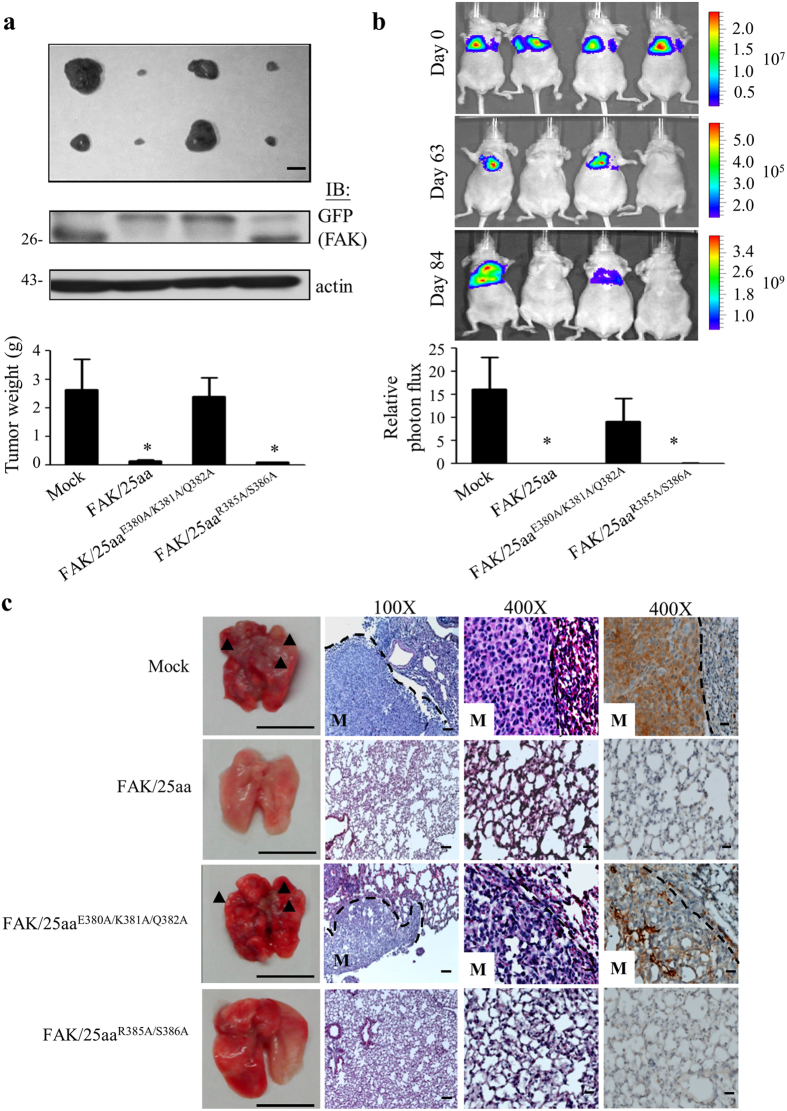
The β4 integrin/FAK complex regulates tumor malignancy *in vivo.* (**a**) MDA-MB-231 cells stably over-expressing GFP-tagged FAK/25aa or its triple (FAK/25aa^E380A/K381A/Q382A^) or double (FAK/25aa^R385A/S386A^) mutant were injected into the 3^rd^ mammary fat pad of nude mice to examine tumor mass and protein expression of the xenograft tumors *in vivo*. Scale bar, 1 cm. The results are shown as the mean ± s.d. *n* = 3 for mock, *n* = 5 for others. *p < 0.05, value was in comparison to mock. The cropped blots were run under the same experimental conditions. The full-length blots are included in Supplementary Fig. S7. (**b**) The above MDA-MB-231 stable cells were injected into the tail veins of nude mice to measure tumor metastasis *in vivo*. The kinetics of breast cancer metastasis to the lung were measured by bioluminescence and representative images are shown at day 0, 63, and 84 after injection. The graph shows the relative photon flux at day 84 after injection. The results are shown as the mean ± s.d. *n* = 3 for mock, *n* = 5 for others. *p < 0.05, value was in comparison to mock. (**c**) Lung metastatic nodules (left column), H&E staining (the second and third columns) and immunohistochemical analysis of GFP protein expression (the fourth column) at lung metastatic sites at day 84 after injection are shown. Lung metastatic nodules are indicated by arrowheads and “M”. Scale bars, 1 cm (left column), 200 μm (the second from left), 100 μm (the third and fourth columns).

To confirm the tumorigenic effects of the β4 integrin/FAK complex we observed in the above *in vitro* studies, we performed *in vivo* tumorigenesis studies by orthotopically injecting MDA-MB-231 cells that stably overexpressed varied FAK/25aa mutants into the mammary fat-pads of nude mice. Mice injected with stably expressing FAK/25aa or FAK/25aa^R385A/S386A^ but not FAK/25aa^E380A/K381A/Q382A^ transfectants of MDA-MB-231 cells showed significant reductions in the size and weight of tumors (Fig. 5a). Protein expression of the transfectants was sustained during tumor growth in the transplanted mice, which reinforces an authentic role for the β4 integrin/FAK complex in breast cancer tumorigenesis.

Subsequently, the involvement of the β4 integrin/FAK complex in breast cancer metastasis was explored by tail vein injection and bioluminescence imaging of various MDA-MB-231 transfectants in nude mice. In comparison with mock and FAK/25aa^E380A/K381A/Q382A^-expressing cells, lung metastatic nodules were less-developed in FAK/25aa- and FAK/25aa^R385A/S386A^-transfectants 84 days after injection in mice (Fig. 5b). In parallel, the histologic analyses confirmed the persistent presence of FAK/25aa and FAK/25aa^R385A/S386A^ expression and its influence on the development of lung metastatic modules (Fig. 5c). These results clearly substantiate a role for the β4 integrin/FAK complex in positively regulating tumorigenesis and metastases in triple-negative breast cancer.

### Concomitant overexpression of β4 integrin and FAK in human triple-negative breast cancer

**Figure 6:**
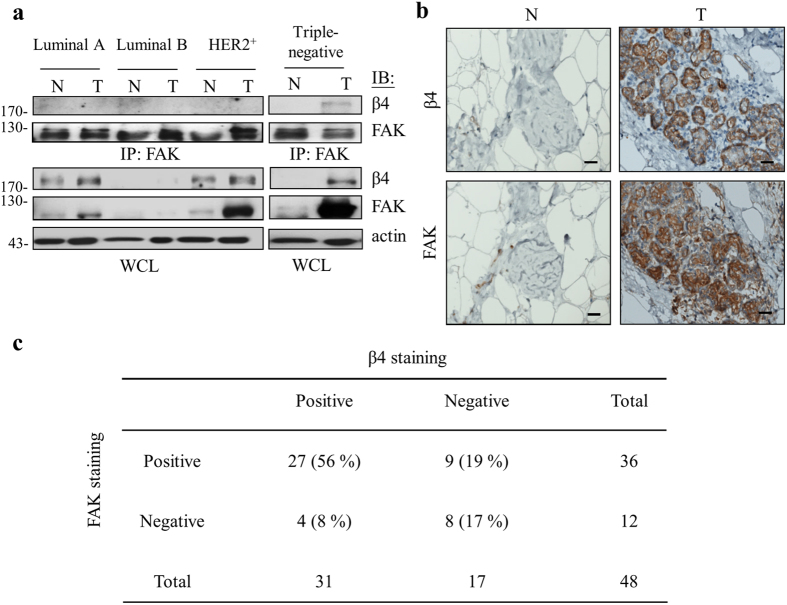
A positive correlation between β4 integrin and FAK expression in patients with triple-negative breast cancer can be therapeutically targeted. (**a**) Homogenized normal (N) and tumor (T) tissue lysates of four patients with luminal A, luminal B, HER2^+^, or triple-negative breast cancer were collected and subjected to immunoprecipitation. FAK co-immunoprecipitated β4 integrin was visualized by Western blot analysis. (**b**) Immunohistochemical staining is shown for β4 integrin and FAK in human triple-negative breast cancer (T) and adjacent non-cancerous breast (N) tissues. Scale bars, 20 μm. (**c**) Spearman’s γ correlation test shows that up-regulation of both β4 integrin and FAK is significantly correlated in 48 human triple-negative breast cancers. *γ* = 0.3772; *p* = 0.0082. All cropped blots were run under the same experimental conditions. The full-length blots are included in Supplementary Fig. S7.

We next analyzed the relationship of β4 integrin and FAK in four subtypes (luminal A, luminal B, HER2^+^, and triple-negative) of human breast cancer (T) and their adjacent non-cancerous counterpart (N) tissues. Consistent with a malignant role for the β4 integrin/FAK complex in triple-negative breast cancer, the β4 integrin co-immunoprecipitated by FAK was predominately associated with triple-negative breast cancer compared to other subtypes (Fig. 6a and Supplementary Fig. S5a). We further employed immunohistochemical staining in malignant triple-negative breast cancer (T) and adjacent non-cancerous (N) tissues to investigate the pathological relevance of β4 integrin and FAK. Our result indicated that concomitant expression of β4 integrin and FAK occurred in triple-negative breast cancer tissues (Fig. 6b and Supplementary Fig. S5b), in that approximately 56% (27 out of 48) of the tumors displayed high levels of both β4 integrin and FAK, whereas approximately 17% (8 out of 48) of the tumors expressed only low levels of both proteins. Statistical results revealed a positive correlation between β4 integrin and FAK expression in these malignant cancer tissues, with a Spearman’s γ correlation of 0.3772 (*p* = 0.0082) (Fig. 6c). In contrast, both proteins were barely detectable in adjacent non-cancerous breast tissues (Fig. 6b). These observations are consistent with previous studies (Figs 1a and 2a) that indicated the simultaneous up-regulation of both β4 integrin and FAK and an interaction between β4 integrin and FAK that is significantly correlated to human malignant triple-negative breast cancer.

## Discussion

**Figure 7:**
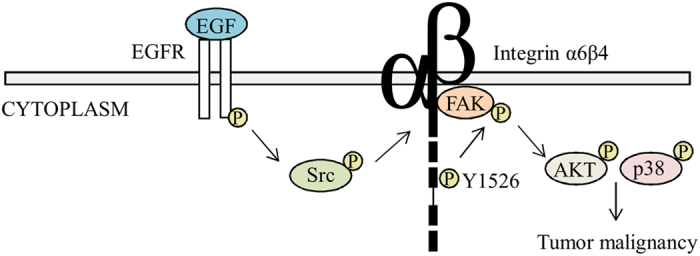
A working model for the mechanism through which the β4 integrin/FAK complex mediates the malignancy of triple-negative breast cancer in an EGF/Src-regulated manner.

In spite of the tight association between integrins and FAK in cell adhesion, the mechanistic details of this signaling axis in diverse pathophysiological functions remains unclear. Our findings regarding the involvement of the β4 integrin/FAK complex in mediating tumor malignancy are summarized in Fig. 7. Here, we provided comprehensive evidence for a physical and functional interaction between β4 integrin and FAK that is mediated by the cytodomain, next to the transmembrane region, of β4 integrin and an 11 amino acid motif that lies ahead of the phospho-Tyr^397^ site of FAK (Fig. 1). Interestingly, this interaction seems to be correlated with the malignant status of breast cancer, supporting a functional relevance for this complex in tumor progression (Fig. 1). Furthermore, the binding of FAK to β4 integrin resulted in an increase of phospho-Tyr^397^ of FAK, which promoted tumor malignancy in concert with the elevated phosphorylation of p38MAPK and AKT (Fig. 3). Therefore, disruption of the β4 integrin/FAK complex led to the reduction of tumorigenicity and metastasis *in vitro* and *in vivo*. More importantly, the β4 integrin/FAK complex was observed to be intimately associated with, and therefore clinical relevant to, triple-negative breast cancer. Our data demonstrates mechanistic details and direct clinical relevance for the β4 integrin/FAK complex in breast cancer malignancy and provides a novel target for use in strategies for intervening in malignant breast cancer.

In addition to the cellular and functional consequences of the β4 integrin/FAK complex in breast cancer progression, numerous clinical pathophysiological studies have confirmed that either β4 integrin or FAK is often overexpressed in human tumor malignancies, including colorectal and gastric cancers^6^,^19^,^32^,^33^. With respect to these findings, we also observed that the β4 integrin/FAK complex was detected exclusively at primary colon cancer sites with high metastatic potential, but not in normal mucosa counterparts (unpublished data). The existence of the β4 integrin/FAK complex in distinct malignant cancers suggests that it might also function as a general oncogenic complex in other tumor types, as it was detected in breast cancer in the present study. Nevertheless, additional genetic analyses will be required to verify the proposed involvement of the β4 integrin/FAK complex in the development of other tumor malignancies.

Microenvironmental cues strongly influence cancer progression. Indeed, we found that the interaction between β4 integrin and FAK occurred in an EGF/Src-dependent manner, indicating the importance of this complex in the dialogue with microenvironmental cues and in the regulation of tumor progression (Fig. 2). However, this interaction may be determined by the tumor microenvironment in relation to tumor stages. When encountering distinct surroundings, it is inevitable that some cancers will fail to form an activated and functioning β4 integrin/FAK complex, despite having a similar expression profile for both molecules, as shown in Fig. 2a and other reports^34^,^35^. Alternatively, varied microenvironmental cues may differentially modulate the expression of β4 integrin and FAK in patients with breast cancer, as indicated by the differences displayed in luminal A, luminal B, HER2^+^, and TNBC (Fig. 6a).

We found that AKT and p38MAPK are involved in β4 integrin/FAK complex-mediated signal transduction (Fig. 3e). These molecules are capable of transmitting tumorigenic signals from the β4 integrin/FAK complex during the regulation of tumor functions, as previously reported^15^,^25^,^29^. These results are consistent with the cellular functions modulated by the β4 integrin/FAK complex, as depicted in Supplementary Fig. S4c and S4d. We hypothesize that FAK serves as a convergence point for both EGFR and β4 integrin signals, to diversify signaling and to initiate p38MAPK and AKT activity during tumor malignancies.

From a therapeutic perspective, we have provided an alternative strategy for targeting cancer therapeutics by using the FAK/25aa peptide to inhibit β4 integrin/FAK complex formation in triple-negative breast cancers (Figs 4 and 5 and our unpublished data). Moreover, the genuine specificity of FAK/25aa peptide was confirmed by cellular and functional experiments (Supplementary Fig. S4a). Although a specific tyrosine kinase inhibitor of FAK significantly represses tumor malignancy, as our unpublished data and others have reported^36^,^37^, off-target effects are a concern due to the potential side effects of clinical therapeutics^36^. Here, the inhibitory efficacy of the FAK/25aa peptide, a non-tyrosine kinase inhibitor of FAK tyrosine activity that targets β4 integrin/FAK complex formation, is supported as a suitable therapeutic strategy. Alternatively, combined targeting with the β4 integrin/FAK complex-targeting agent with inhibitors of signaling effectors along the transduction pathway that are identified in this study may exhibit synergistic effects that inhibit tumor malignancies.

In conclusion, we have demonstrated that β4 integrin and FAK physically and functionally interact with each other *in vitro* and *in vivo*. This interaction is modulated by EGF/Src signaling, which triggers a tyrosine phosphorylation cascade that regulates tumor proliferation, migration, and invasion as well as metastasis *in vitro* and *in vivo*. Several signaling mediators, i.e., AKT and p38MAPK, may play pivotal roles in β4 integrin/FAK-mediated tumor functions. Our results demonstrate a crucial signaling module involved in the regulation of malignancy in triple-negative breast cancer and a novel target for future interventions and anti-cancer therapeutics.

## Methods

### Materials

Plasmid DNA construction, antibodies and reagents are described in Supplementary Methods.

### Human tissue samples

A tissue microarray containing 48 paraffin-embedded human triple-negative breast cancer samples (BRC964) was purchased from Pantomics, Inc. (Richmond, CA). The surgical specimens of primary cancerous breast tissues and surrounding non-cancerous breast tissues that were used for Western blotting and immunoprecipitation analysis were obtained from four patients who were not given preoperative chemotherapy and who had undergone resection with curative intent between September 2010 and December 2013 at National Taiwan University Hospital (Taipei, Taiwan) (Supplementary Fig. S5a). All tissues were collected with informed consent according to the Institutional Review Board of National Taiwan University Hospital (Taipei, Taiwan). All experimental protocols in this study were approved by the National Taiwan University Hospital Research Ethics Committee and were carried out in accordance with the Institutional Review Board of National Taiwan University Hospital (Taipei, Taiwan).

### Immunohistochemical analysis

Paraffin-embedded human cancer samples were sectioned and stained after antigen retrieval using primary antibodies against FAK (C20, 1:200) and β4 integrin (H101, 1:200), followed by a biotinylated and peroxidase-conjugated secondary antibody. The sections were processed by using a DAB immunostaining assay kit (DAKO, Glostrup, Denmark) according to the manufacturer’s instructions. The samples were further counterstained with hematoxylin before mounting on coverslips. They were then examined under a fluorescence microscope (Model M1, Zeiss, Germany) with a 10× or 40× objective lens, and the images were captured using a CCD camera (DP71, Olympus, Japan). The level of staining was scored by Quick-score (Q-score) method based on the staining intensity and the percentage of tumor cells with positive staining. The staining intensity was scored as 0, 1, 2, or 3 corresponding to negative, weak, moderate, or strong, respectively. The percentage of tumor cells positively stained was scored as 0, 1, 2, 3, or 4 corresponding to 0%, 1–25%, 26–50%, 51–75%, or 76–100%, respectively. The Q-score of each tissue sample was the sum of the staining intensity and the percentage of tumor cells with positive staining. The score range was from 0 to 7. A Q-score >2 was defined as overexpressed or positive expression, and a Q-score <2 was defined as normal or negative expression.

### Cell culture and transfection

MCF7 human breast cancer, MDA-MB-231 human breast cancer, MDA-MB-435 human melanoma, and 293T human epithelial kidney cell lines were maintained in Dulbecco’s modified Eagle’s medium (DMEM) supplemented with 10% fetal bovine serum (FBS) (Invitrogen). MCF10A normal mammary epithelial cells were cultured in Medium-171 containing MEGS (Life Technologies). Mouse fibroblast NIH3T3 cells were cultured in DMEM medium containing 10% calf serum (Invitrogen). All cells were incubated in a 37 °C humidified 5% CO_2_ incubator. Cells were transfected with mammalian expression plasmids, as indicated, using Lipofectamine 2000^TM^ transfection reagent (Invitrogen) according to the manufacturer’s instructions. Experiments were conducted 24–48 h after transfection.

### Immunofluorescence staining

Cells were processed for immunofluorescence staining as previously described^37^. In the EGF-stimulated condition, cells were treated with 10 ng/ml EGF for 10 min after overnight serum starvation. The primary antibodies used were polyclonal anti-FAK (C20, 1:200) and monoclonal anti-β4 integrin (3E1, 1:200). Alexa Fluor 488-conjugated goat anti-rabbit IgG and Texas Red-conjugated goat anti-mouse IgG were used as the secondary antibodies. Cell nuclei were stained with DAPI for 5 min at RT. Cells were then mounted using a SlowFade® Light Antifade Kit (Molecular Probes, Inc.) and examined under a confocal laser scanning microscope (LSM 780, ZEISS) with a 63× objective lens.

### Western blotting and immunoprecipitation

Various plasmid-transfected or pharmacologically treated cells or tissue samples were homogenized and extracted for Western blot analyses as previously described^38^. About 10–20 μg whole cell lysate were used for Western blotting. A 1 mg/ml concentration of total protein from cell lysates was employed for immunoprecipitation. Some extracts subjected to immunoprecipitation were incubated with antibodies, as indicated, for 4 h at 4 °C, followed by incubation for 4 h or overnight with protein A-Sepharose 4B or protein G-Sepharose beads (Sigma-Aldrich) before proceeding to Western blot analysis. Each experiment was repeated at least three independent times and the information from the densitometric analysis is indicated in Supplementary Fig. S6.

### Preparation of recombinant fusion proteins

These experiment were performed as previously described^37^, with the following modifications. The constructs were transformed into a BL21 strain and grown at 37 °C until at an optical density at 600 nm of 0.6. They were then induced with 1 mM isopropyl-β-thiogalactopyranoside overnight at 26 °C. Subsequently, cells were pelleted and resuspended with PBS following sonication with a Misonix sonicator 3000. Then, Triton X-100 (1%) was added and cells were incubated on ice for 1 h. The lysates were clarified by centrifugation and then immobilized on GST-agarose beads (Sigma-Aldrich) or Nickel-nitrilotriacetic agarose (Ni-NTA, Qiagen) for 6 h at 4 °C. Finally, the beads were washed and then eluted.

### Lentivirus production and infection

Lentiviruses encoding ITGB4 small-hairpin RNAs (shRNA) or LUCIFERASE small-hairpin RNA was obtained from the TRC lentiviral shRNA library in the National RNAi Core Facility of Academia Sinica, Taiwan. The targeting sequences of specific shRNAs are shown as follows: ITGB4 shRNA (clone ID: TRCN0000057769) 5′-CCCATGAAGAAAGTGCTGGTT-3′, ITGB4 shRNA (clone ID: TRCN0000057771) 5′-GAGGGTGTCATCACCATTGAA-3′, and LUCIFERASE shRNA (clone ID: TRCN0000072246) 5′-CAAATCACAGAATCGTCGTAT-3′. Production of lentiviruses was performed according to the guidelines of the National RNAi Core Facility of Academia Sinica.

### BrdU incorporation assay

At 24 h after transfection, 2 × 10^4^ cells were serum starved for 24 h. Cells were then washed twice with DMEM and incubated for 16 h in DMEM plus 10% FBS and 100 μM BrdU (Sigma-Aldrich). After that, cells were fixed, permeabilized, treated with DNase I, and processed for immunofluorescence staining with anti-BrdU (1:200, Sigma-Aldrich) antibody, as described previously^39^, with a few modifications. Cells were then counted in multiple fields and scored for BrdU-positive staining in each independent experiment.

### Anchorage-independent growth in soft agar assay

Experiments were performed as previously described^39^, with the following modifications. A total of 5 × 10^4^ cells were seeded in 0.3% agar in DMEM plus 10% FBS and EGF (10 ng/ml) over the bottom 0.6% agar layer in DMEM. After incubation for 14 days, the number of colonies was scored.

### Modified Boyden chamber cell migration assay

A Neuro Probe 48-well chemotaxis Boyden chamber (Cabin John, MD) was used. A total of 5 × 10^4^ cells were allowed to migrate toward 10% FBS, EGF (10 ng/ml) in DMEM, used as the chemoattractant in the lower wells for 6 h. Finally, cells on the upper side of the polycarbonate membrane were removed and the bottom-side cells were fixed with methanol for 8 min and stained with crystal violet (Sigma-Aldrich). The migrated cells were counted from five randomly selected fields of each well.

### Matrigel invasion assay

BD BioCoat^TM^ Matrigel^TM^ invasion chambers were rehydrated by DMEM for 2 h. After removing the DMEM, EGF (10 ng/ml), and 10% FBS in DMEM was used as a chemoattractant in the lower wells of the invasion chamber. A total of 5 × 10^4^ cells in DMEM were placed into the upper chamber. Cells were incubated for 20 h to allow them to invade into the Matrigel. Subsequently, cells were fixed with 4% paraformaldehyde for 15 min and stained with crystal violet (Sigma-Aldrich). The number of invaded cells was counted from five randomly selected fields in each well.

### Modeling tumorigenesis and metastasis *in vivo*

All mouse experiments were approved by the Institutional Animal Care and Use Committee, National Taiwan University (Taipei, Taiwan). All experimental procedures were performed in accordance with the protocols and the ethical regulations approved by the Institutional Animal Care and Use Committees of National Taiwan University (Taipei, Taiwan). Female *nu*/*nu* mice were purchased from the National Laboratory Animal Center (Taipei, Taiwan). For tumorigenesis, 1 × 10^6^ MDA-MB-231 stably transfected pools expressing GFP-tagged FAK/25aa, its triple (FAK/25aa^E380A/K381A/Q382A^) or double (FAK/25aa^R385A/S386A^) mutant, or a mock transfected control that had been selected by 500 μg/ml G418 for 2 weeks were injected into the 3^rd^ mammary fat pad of eight-week-old female *nu*/*nu* mice. Stably transfected pools of each construct were injected in 100 μl PBS. Tumor volumes and numbers were measured at 12 weeks after injection and then excised, photographed, and weighted. For tumor metastasis, 1 × 10^6^ MDA-MB-231 cells, described above, that were labeled with luciferase by lentiviral infection were resuspended in 100 μl PBS and injected into the tail vein of six-week-old female *nu*/*nu* mice. Lung metastasis was monitored by bioluminescent imaging using an IVIS spectrum imaging system. Lung metastasis was measured on the respective day after injection.

### Statistical analysis

Student’s t-test was used for statistical analyses. The data in this study are presented as the mean and error bars represent the standard deviation. The data were acquired from at least three independent experiments. *p < 0.05 was considered significant differences among the experimental groups. Spearman’s γ correlation test was used to assess the relationship between β4 integrin and FAK.

## Additional Information

**How to cite this article**: Tai, Y.-L. *et al.* An EGFR/Src-dependent β4 integrin/FAK complex contributes to malignancy of breast cancer. *Sci. Rep.* **5**, 16408; doi: 10.1038/srep16408 (2015).

## Supplementary Material

Supplementary Information
