# Supplementary material for: An EGFR/Src-dependent β4 integrin/FAK complex contributes to malignancy of breast cancer
Source: Sci Rep. 2015 Nov 9;5:16408. doi: 10.1038/srep16408 (PMC4637903; doi:10.1038/srep16408)

An EGFR/Src-dependent β4 integrin/FAK complex contributes to malignancy of breast cancer

Yu-Ling Tai[1], Pei-Yu Chu[1], I-Rue Lai[2], Ming-Yang Wang[3], Hui-Yuan Tseng[1], Jun-Lin Guan[4], Jun-Yang Liou[5], and Tang-Long Shen[1, 6, *]

[1]Department of Plant Pathology and Microbiology, National Taiwan University, Taipei 10617, Taiwan, [2]Department of Anatomy and Cell Biology, College of Medicine, National Taiwan University, Taipei 10051, Taiwan, [3]Department of Surgery, National Taiwan University Hospital, Taipei 10048, Taiwan, [4]Department of Cancer Biology, University of Cincinnati College of Medicine, Cincinnati, OH 45267, USA, [5]Institute of Cellular and System Medicine, National Health Research Institutes, Zhunan, Miaoli County 35053, Taiwan, [6]Center for Biotechnology, National Taiwan University, Taipei 10617, Taiwan

***Correspondence:**

Tang-Long Shen, Department of Plant Pathology and Microbiology, National Taiwan University, No. 1, Sec. 4, Roosevelt Road, Taipei, 10617, Taiwan. Phone: 886-2-33664998; Fax: 886-2-23636490; E-mail: shentl@ntu.edu.tw

Supplementary Information

Supplementary Methods

Plasmid DNA construction. Several mammalian expression vectors, including pKH3 (containing an amino terminal triple HA tag), pHAN (containing an amino terminal Myc-6xHis tag), pcDNA-3.1-Myc/6xHis (containing a carboxyl terminal Myc-6xHis tag; BD Biosciences), pDH-GST (containing an amino terminal GST tag), and pEGFP (containing an amino or carboxyl terminal GFP tag; BD Biosciences), were employed for related FAK, Src, and β4 integrin constructs used in this study. Several bacterial expression vectors, including pGEX-2T (containing an amino terminal GST tag) and pET 28a (containing an amino terminal His tag), were employed for related β4 integrin and FAK constructs used in this study. The pKH3-FAK and its various mutants, such as pKH3-FAK^{Y397F} (Tyr^{397} converted to Phe of full length FAK), pKH3-FAK/KD (kinase dead), pKH3-FAK/ΔN (deleted first 400 amino acids), pKH3–FAK/N400 (the first 400 amino acids), pKH3-FAK/N375 (the first 375 amino acids), and pKH3-FRNK, as well as their Myc-6xHis tagged expression pHAN vector counterparts, have been described previously[1] . pKH3-FAK/N386 (the first 386 amino acids), pKH3-FAK/387C (deleted first 386 amino acids), pKH3-FAK^{E380A},

pKH3-FAK^{K381A}, pKH3-FAK^{Q382A}, pKH3-FAK^{G383A}, pKH3-FAK^{R385A}, pKH3-FAK^{S386A}, pKH3-FAK$^{E380A/K381A/Q382A}$, pKH3-FAK$^{R385A/S386A}$, pDH-GST-FAK/25aa, pEGFP-FAK/25aa, pEGFP-FAK/25aa$^{E380A/K381A/Q382A}$, and pEGFP-FAK/25aa$^{R385A/S386A}$ were constructed by PCR and subcloned into the indicated vectors. The point mutants within the identified 25-amino acid motif of FAK were generated by an overlapping PCR mutagenesis approach as described previously[1]. The pcDNA3.1-Zeo-β4 integrin and α6 integrin plasmids were used as templates to construct related β4 integrin mutants as described previously[2]. In general, DNA subcloning and PCR were used to generate pcDNA-3.1-Myc/6xHis-β4 integrin, pcDNA-3.1-Myc/6xHis-β4 integrin^{Y1422F}, pcDNA-3.1-Myc/6xHis-β4 integrin^{Y1440F}, pcDNA-3.1-Myc/6xHis-β4 integrin^{Y1494F}, pcDNA-3.1-Myc/6xHis-β4 integrin^{Y1526F}, pcDNA-3.1-Myc/6xHis-β4 integrin^{Y1642F}, pEGFP-β4 integrin, pEGFP-β4/∆FNIII(L-3-4) (deleted from the linker to the carboxyl terminus), pEGFP-β4/∆FNIII(3-4) (deleted after linker to the carboxyl-terminus), pEGFP-β4/∆cyto (deleted the entire cytoplasmic tail), and pEGFP-β4/tailless (deleted the whole intracellular domain). Likewise, pKH3-β4/cytodomain (the 796th to 1190th amino acid), pKH3-β4/FN III (1-2), pKH3-β4/FN III (1-2-L), and pKH3-β4/FN III (3-4-C) fragments were generated by PCR into pKH3 vector by BamHI. The pKH3-Src, pKH3-Src^{Y527F}, and pKH3-Src^{K295M} were constructed by PCR and cloned

into pkH3 vector. All resulting constructs were confirmed by sequencing and protein expression was verified by Western blot analysis.

Antibodies and reagents. Antibodies used were anti-EGFR (1005), anti-FAK (C20), anti-Myc (9E10), anti-HA (Y-11), anti-HA (12CA5), anti-GFP (N16), anti-GFP (B2), anti-β4 integrin (H101), anti-β1 integrin (M106), anti-Src (N16), anti-phospho-tyrosine (PY99), and anti-tubulin, which were purchased from Santa Cruz Biotechnology, Inc. (Santa Cruz, CA, USA); anti-pY1526 was obtained from ECM Biosciences; anti-ERK1/2, anti-phospho-ERK (pT202/pY204), anti-p38 MAP kinase (N20), anti-phospho-p38 MAP Kinase (Thr180/Tyr182), anti-SAPK/JNK, anti-phospho-SAPK/JNK (Thr183/Tyr185), anti-AKT, anti-phospho-AKT (S473), and anti-phospho-Stat3 (Y705) were obtained from Cell Signaling (Danvers, MA); anti-β4 integrin (3E1) and anti-actin were bought from Chemicon; anti-polyhistidine, anti-phospho-FAK (Y397) and anti-phospho-Src (Y418) were obtained from Invitrogen; and anti-GST and anti-His were obtained from Upstate (Charlottesville, Va.) and Sigma-Aldrich, respectively. The anti-phospho-paxillin (Tyr118) and anti-paxillin were bought from BD (Becton, Dickinson and Company). The anti-EGFR (Tyr1173) was bought from Millipore. The anti-Stat3 antibody was a gift from Dr. Chien-Kuo Lee (Institute of Immunology, National Taiwan University). Inhibitors for

EGFR (AG1478), Src kinase (PP2), PP3, p38 (SB203580), AKT (AKT inhibitor VI) and PI3 kinase (LY294002) were purchased from Calbiochem (San Diego, CA, USA). EGF was bought from BD (Becton, Dickinson and Company).

Far-Western blotting assay. Immunoprecipitated or purified recombinant β4 integrin was separated by SDS-PAGE and then transferred to a nitrocellulose membrane. Following Ponceau S staining to ensure transfer, the membrane was first incubated with AC buffer (10% Glycerol, 100 mM NaCl, 200 mM Tris, pH 7.5, 1 mM EDTA, 0.1% Tween-20, 2% milk powder (W/V), 1 mM DTT, and 8 M guanidine–HCl), followed by gradually and sequentially reducing the guanidine–HCl concentration until it reached zero to renature the proteins on the membrane. Next, the membrane was blocked for 1 h at room temperature in TBS (containing 0.1% Tween-20 and 5% BSA) and incubated overnight at 4 ºC with 1 μg/ml recombinant 6x-histidine fusion proteins that were previously adsorbed to Nickel-nitrilotriacetic agarose (Ni-NTA, Qiagen) in 0.5% BSA. Subsequently, anti-polyhistidine antibody was used to blot the membrane for 1 h at RT and the Western analysis was continued as described above.

Supplementary References

1. Shen, T.L., Han, D.C. & Guan, J.L. Association of Grb7 with phosphoinositides and its role in the regulation of cell migration. *J. Biol. Chem.* **277**, 29069-77 (2002).
2. Abdel-Ghany, M., Cheng, H.C., Elble, R.C. & Pauli, B.U. Focal adhesion kinase activated by beta(4) integrin ligation to mCLCA1 mediates early metastatic growth. *J. Biol. Chem.* **277**, 34391-400 (2002).

Supplementary Figure Legends

Supplementary Figure S1. Direct interaction between the cytodomain of β4 integrin and an amino-terminal linker within FAK. (a) Cell lysates from MDA-MB-231 cells were incubated with the His-tagged FAK amino- (i.e., FAK/N375 and FAK/N400) or carboxyl- (i.e., FRNK) recombinant proteins (shown on the bottom by Coomassie blue staining). Subsequently, His-tagged proteins were pulled down by Ni-conjugated beads, followed by Western blot analysis with anti-β4 integrin antibody. (b) In the far-Western assay, immunoprecipitated β4 integrin, obtained from MDA-MB-231 endogenous β4 integrin (endogenous) or ectopic expression of Myc-tagged β4 integrin (exogenous), was subjected to SDS-PAGE and then transferred onto nitrocellulose membranes. β4 integrin protein that was renatured by removing SDS was incubated with the purified His-tagged FAK amino- or carboxyl- recombinant proteins, as indicated (shown on the bottom by Coomassie blue staining), then Western blot analysis with anti-His antibody against FAK proteins was used to

detect the capability of FAK to bind directly to β4 integrin. (c) A summary of various FAK truncated mutants that were capable of interacting with β4 integrin is listed. (d) HEK293 cells were co-transfected with Myc-tagged integrin α6β4 and HA-tagged full-length FAK or its amino-terminal fragments, i.e., FAK/N386 and FAK/N375. Cell lysates were immunoprecipitated by anti-HA antibody against FAK or its mutants, and the co-immunoprecipitated β4 integrin was visualized by anti-Myc antibody. The results indicated that the 376th to the 386th amino acids within FAK were capable of binding β4 integrin. HEK293 cells were co-transfected with Myc-tagged integrin α6β4 and HA-tagged full-length FAK or its amino-terminal deletion mutants (i.e., FAK/ΔN, FRNK and FAK/387C) or amino-terminal fragments (i.e., FAK/N400 and FAK/N386). Cell lysates were collected and immunoprecipitated by an anti-Myc antibody against β4 integrin (e) or an anti-HA antibody against FAK (f). Co-immunoprecipitated β4 integrin or FAK fragments were visualized by anti-Myc or anti-HA antibodies, respectively. (g) HEK293 cells were co-transfected with HA-tagged FAK, Myc-tagged α6 integrin and GFP-tagged full-length β4 integrin or its various cytoplasmic domain deletion mutants to determine the FAK binding domain within β4 integrin. Cell lysates were collected and immunoprecipitated by an anti-HA antibody against FAK, and co-immunoprecipitated β4 integrin fragments were visualized by an anti-GFP antibody. (h) A summary of various β4 integrin

truncated mutants that were capable of interaction with FAK is listed. β4 integrin truncated mutants are listed, including cytodomain (containing the region between transmembrane domain, TM, and FNIII repeat 1), FNIII(1-2) (containing FNIII repeats 1 and 2), FNIII(1-2-L) (containing FNIII repeats 1 and 2 and linker segment), FNIII(3-4-C) (containing FNIII repeats 3, 4 and carboxyl end), and those that lacked either the whole cytoplasmic domain (designated as △cyto), the FNIII repeats 3 and 4 (designated as △FNIII 3-4), or the linker region to the carboxyl end (designated as △FNIII L-3-4).

Supplementary Figure S2. EGF/Src signaling leads to increased tyrosine phosphorylation of β4 integrin and the formation of the β4 integrin/FAK complex. (a) MCF7 cells were first serum starved overnight and then stimulated with or without EGF (10 ng/ml) to examine the effect on the tyrosine phosphorylation of β4 integrin and FAK and the interaction between β4 integrin and FAK. Cell lysates were collected and immunoprecipitated by an anti-β4 integrin antibody. Immune complexes were analyzed by Western blot analysis with an anti-phospho-Tyr1526 antibody against β4 integrin phosphorylation, or anti-β4 integrin and anti-FAK antibodies. (b) Serum-starved MDA-MB-231 cells were treated with EGF (10 ng/ml) to examine the co-localization of β4 integrin (green) and FAK (red) by

immunofluorescent staining. Arrows indicate the distribution of FAK at focal adhesions and/or on the peripheral plasma membrane. Arrowheads indicate the localization of β4 integrin on the plasma membrane. Scale bars, 20 μm.

Supplementary Figure S3. Effects of β4 integrin/tailless on the formation of the β4 integrin/FAK complex. In the immunoprecipitation assay, the phosphorylation level of FAK at Tyr^{397} and the co-immunoprecipitated FAK by anti-β4 integrin antibody were detected in MDA-MB-231 cells that expressed increasing amounts of β4 integrin/tailless mutant. The MDA-MB-231 cells described above were employed for immunoprecipitation by an anti-α6 integrin antibody to detect the difference in α6 integrin binding ability with the β4 integrin/tailless mutant and full-length β4 integrin.

Supplementary Figure S4. Effects of the β4 integrin/FAK complex on tumor functions *in vitro*. (a) No effects of the β4 integrin/FAK complex in NIH3T3 cells were observed. NIH3T3 cells expressing GFP-tagged FAK/25aa or FRNK were subjected to cell migration assay toward EGF (50 ng/ml) or fibronectin (10 μg/ml) in a modified Boyden chamber assay. DMEM was used as a negative control. (b) MDA-MB-231 cells expressing HA-tagged FAK/25aa, FRNK or both (FAK/25aa+FRNK) were subjected to analyze cell migration toward EGF (20 ng/ml)

in a modified Boyden chamber assay. (c) MDA-MB-231 cells expressing GFP (mock) or GFP-tagged FAK/25aa were subjected to a soft agar assay in the presence of EGF (10 ng/ml) with p38 inhibitor (10 μM SB203580) or AKT inhibitor (10 μM AKT-in) to examine the capability for anchorage-independent growth. (d) MDA-MB-231 cells expressing GFP-tagged FAK/25aa or its triple (FAK/25aa$^{E380A/K381A/Q382A}$) or double (FAK/25aa$^{R385A/S386A}$) mutant were treated with p38 inhibitor (10 μM SB203580) or AKT inhibitor (10 μM AKT-in) and subjected to analysis of cell migration toward EGF (10 ng/ml) in a modified Boyden chamber assay. Data in (a-d) are shown as the mean, and error bars represented the standard deviation from at least three independent experiments. *, $p < 0.05$, value is in comparison to the corresponding mock control.

Supplementary Figure S5. Immunohistochemistry of β4 integrin or FAK expression in human triple-negative breast cancer tissues. (a) Summary of clinicopathological characteristics of the patients with breast cancer. (b) The intensity of β4 integrin (top) or FAK (bottom) staining in tissues is shown as indicated. Staining intensity was scored as 0 (negative), +1 (weak), +2 (moderate), or +3 (strong). Scale bars, 20 μm.

Supplementary Figure S6. The densitometric analysis shows the relative

quantification of band intensities, normalized to respective controls, for Figures 1a, 1c-1f, 2a-2e, 3a-3c, 3e and 4a. Student's t-test was used for statistical analysis. The data are represented as the mean and error bars represent the standard deviation. The data were acquired from at least three independent experiments. *, $p < 0.05$ was considered a significant difference among the experimental groups.

Supplementary Figure S7. Full-length blots for Figures 1a, 1c-1f, 2a-2e, 3a-3e, 4a, 5a, and 6a.

Supplementary Figure S1

Tai et. al.

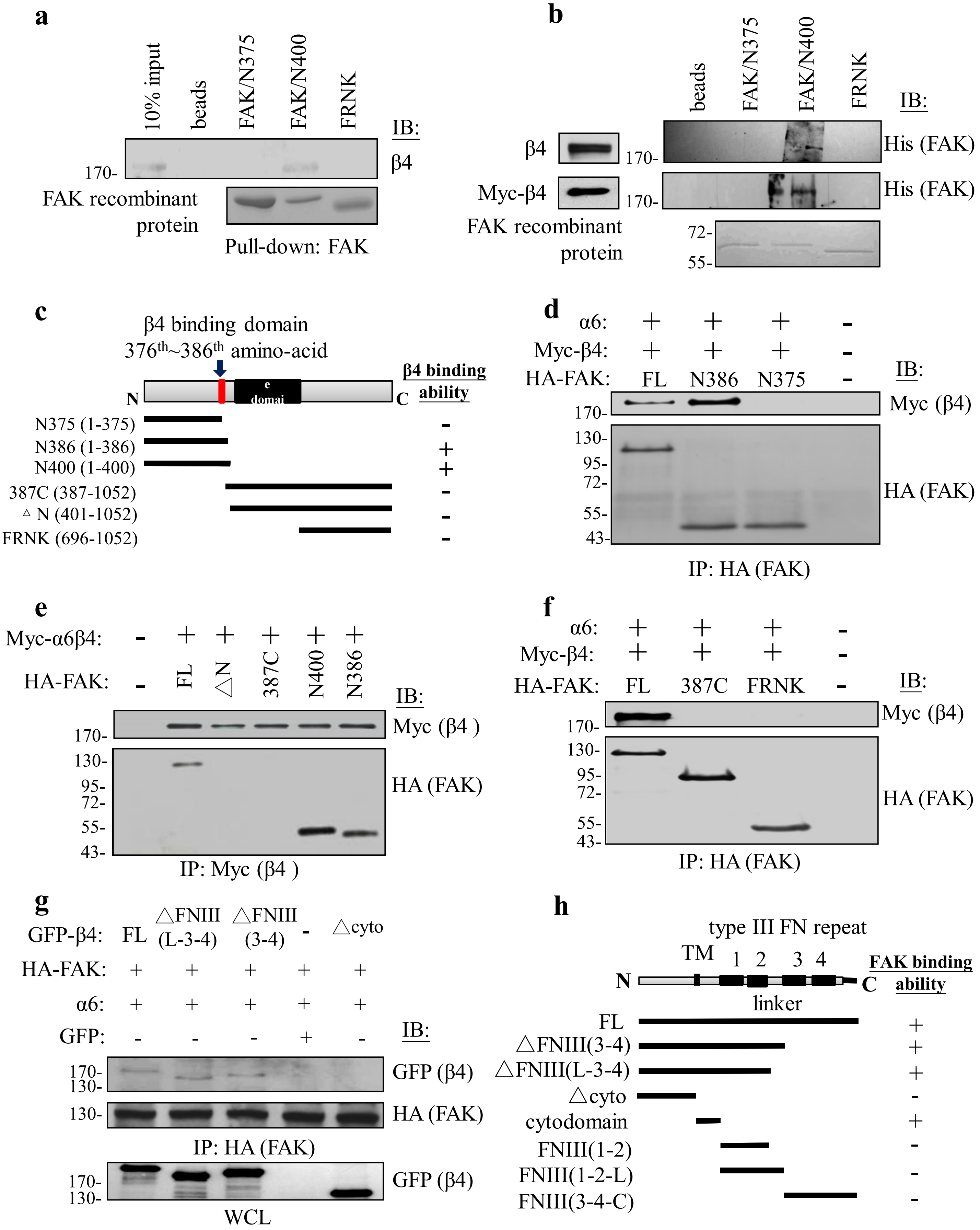

Supplementary Figure S2

Tai et. al.

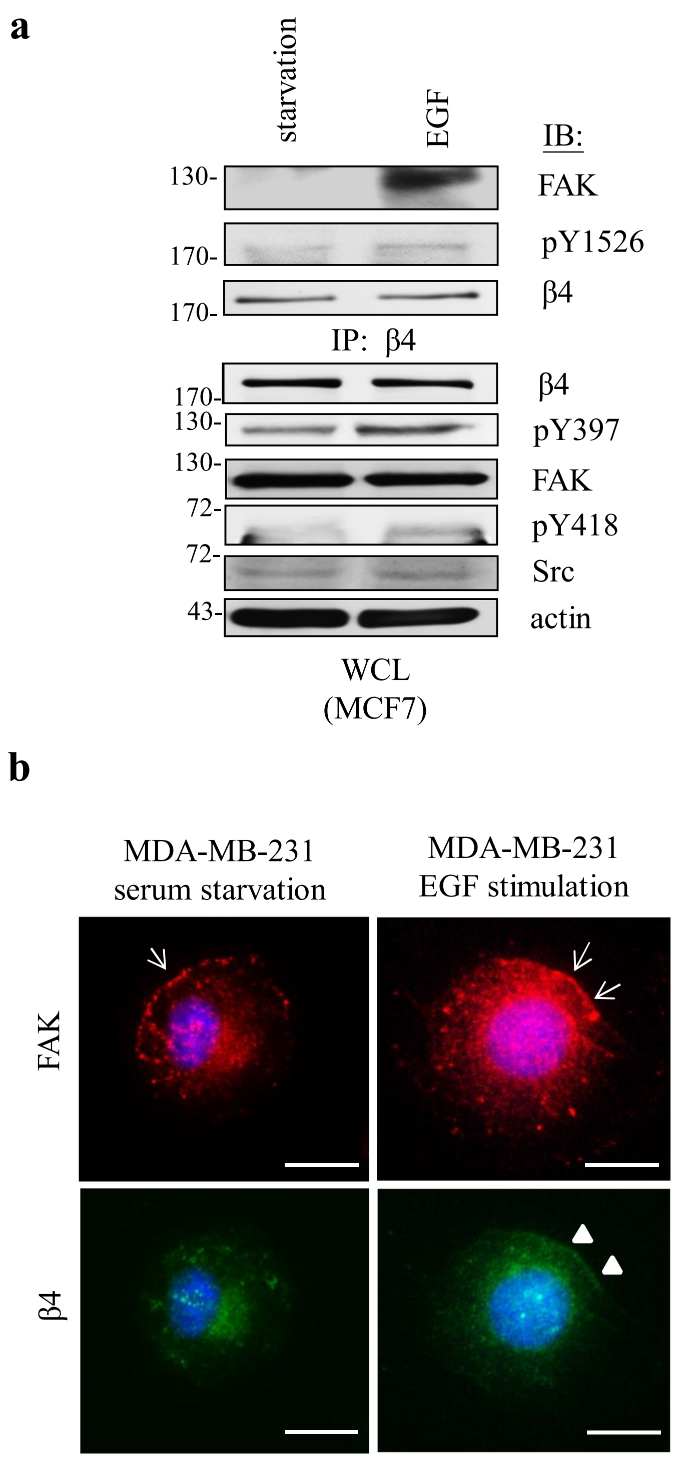

Supplementary Figure S3

Tai et. al.

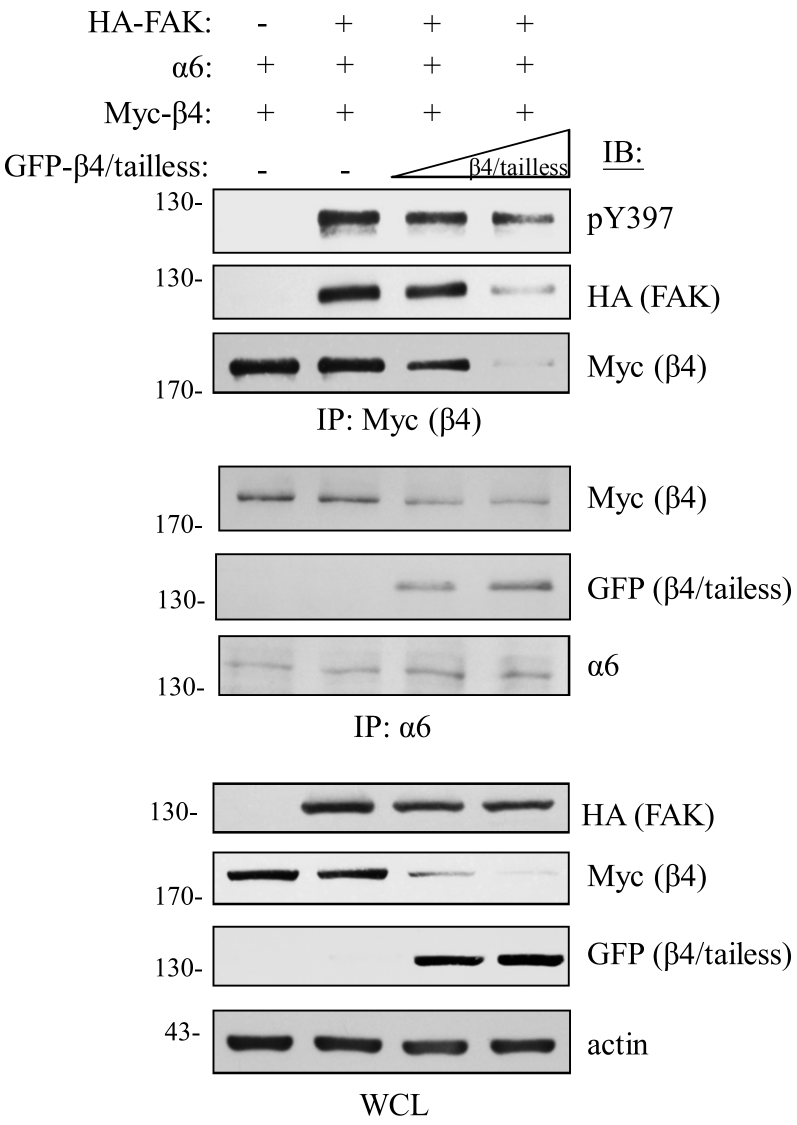

Supplementary Figure S4

Tai et. al.

a

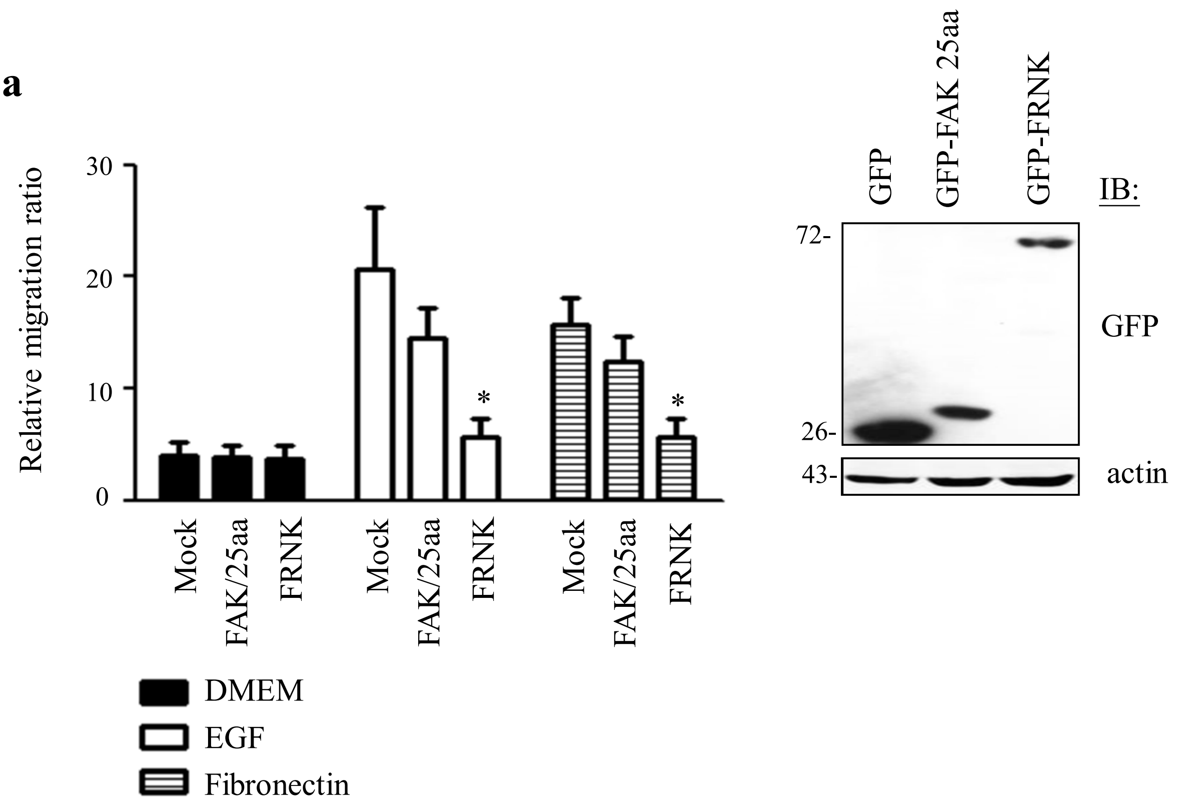

b

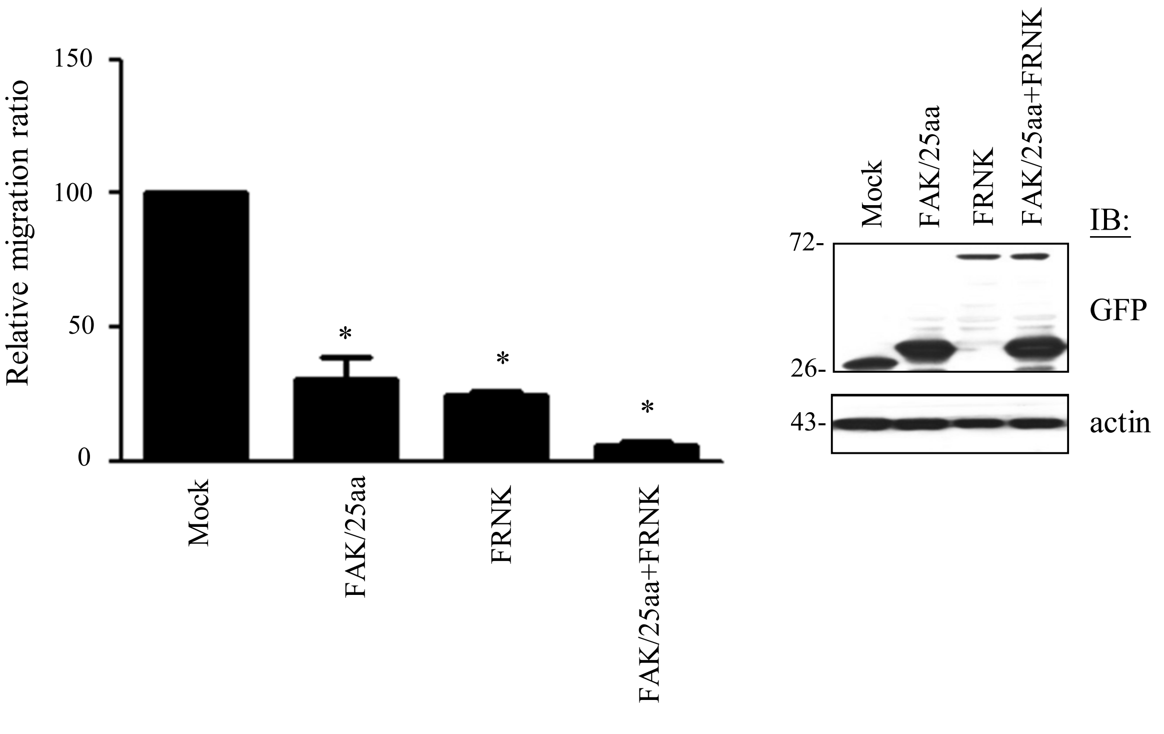

Supplementary Figure S4

Tai et. al.

c

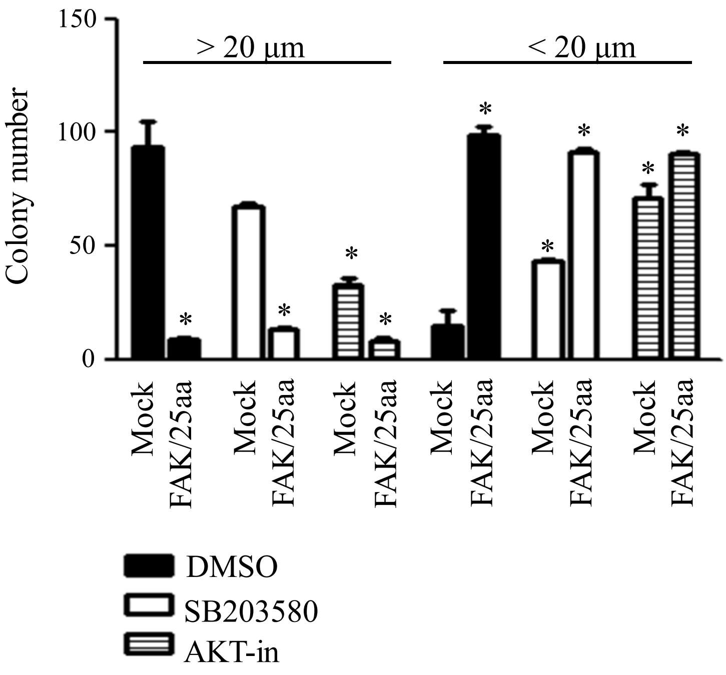

d

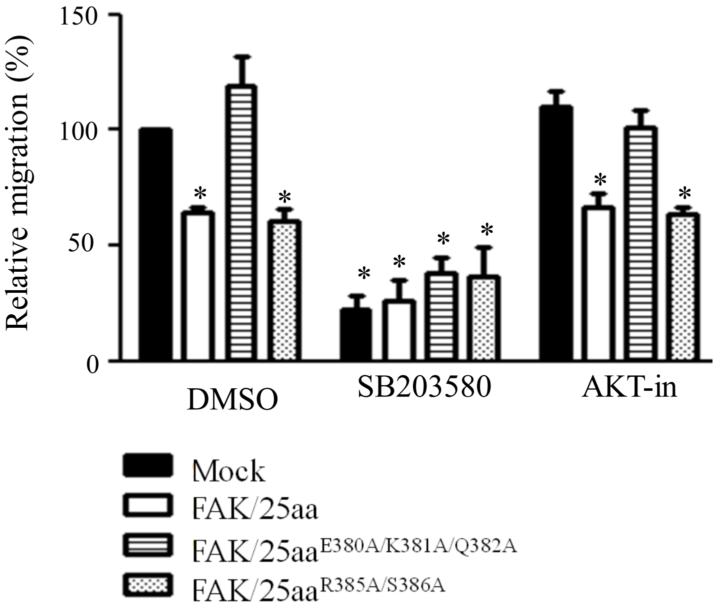

Supplementary Figure S5

Tai et. al.

a

Subtype	Type of carcinoma	Pathology Grade	ER	PR	Neu
Luminal A	Invasive ductal carcinoma	I	>95%	>95%	-
Luminal B	Invasive lobular carcinoma	III	100%	60%	3+
HER2^{+}	Invasive ductal carcinoma	III	-	-	3+
Triple-negative	Invasive ductal carcinoma	II	-	-	-

b

The intensity of β4 integrin staining:

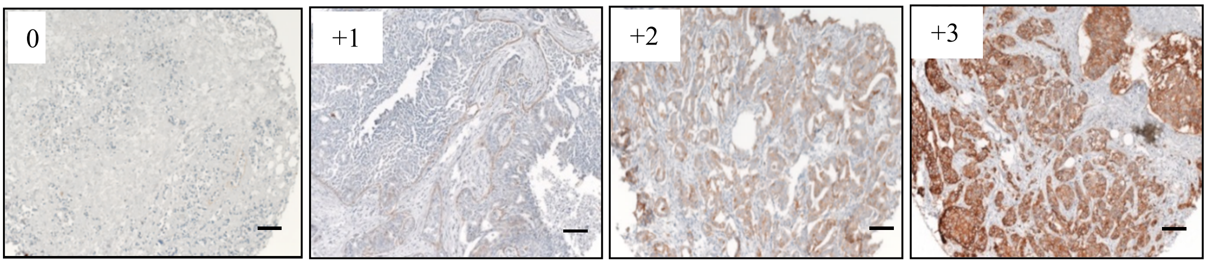

The intensity of FAK staining:

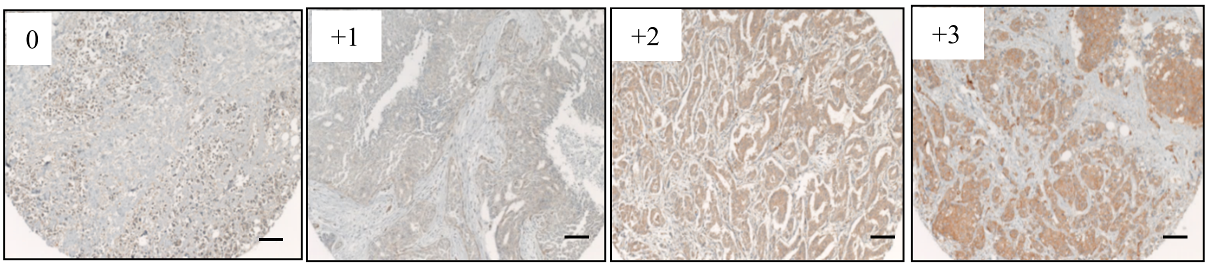

Supplementary Figure S6

Tai et. al.

Relative to Figure 1a

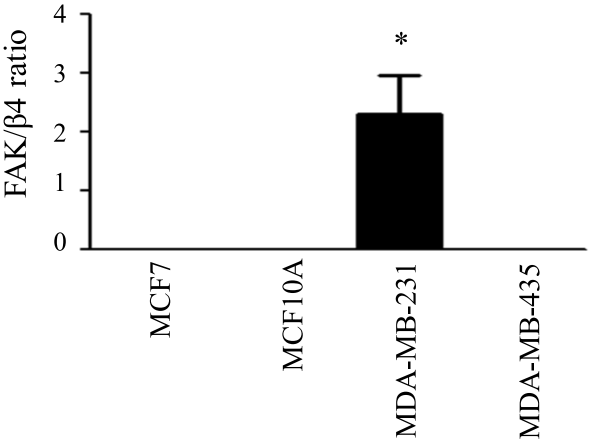

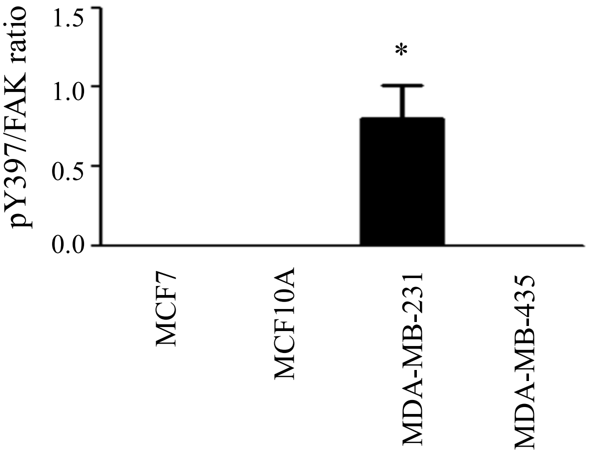

Relative to Figure 1c

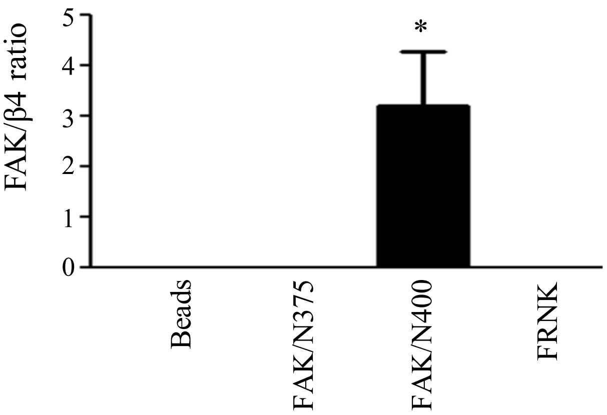

Relative to Figure 1d

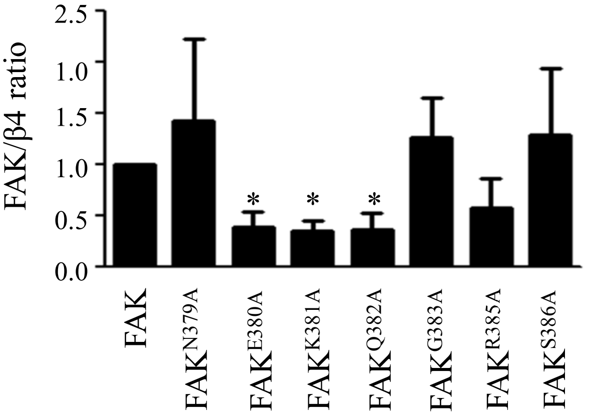

Relative to Figure 1e

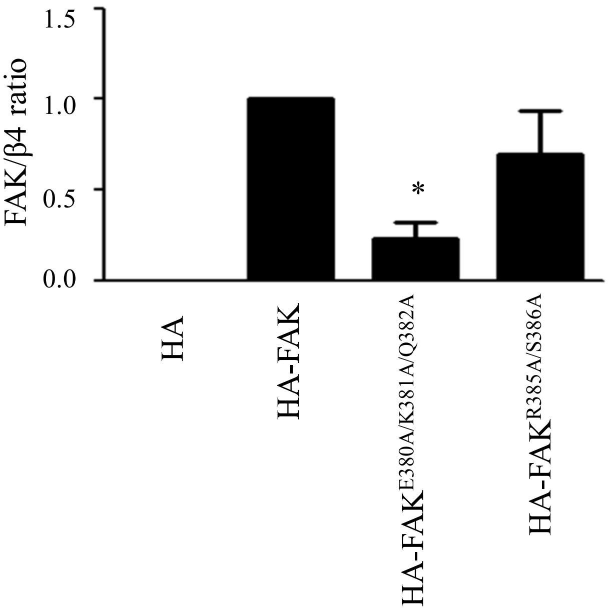

Relative to Figure 1f

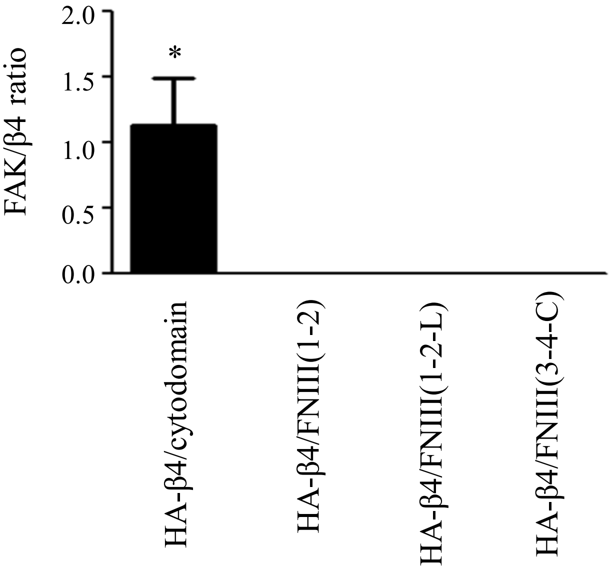

Supplementary Figure S6

Tai et. al.

Relative to Figure 2a

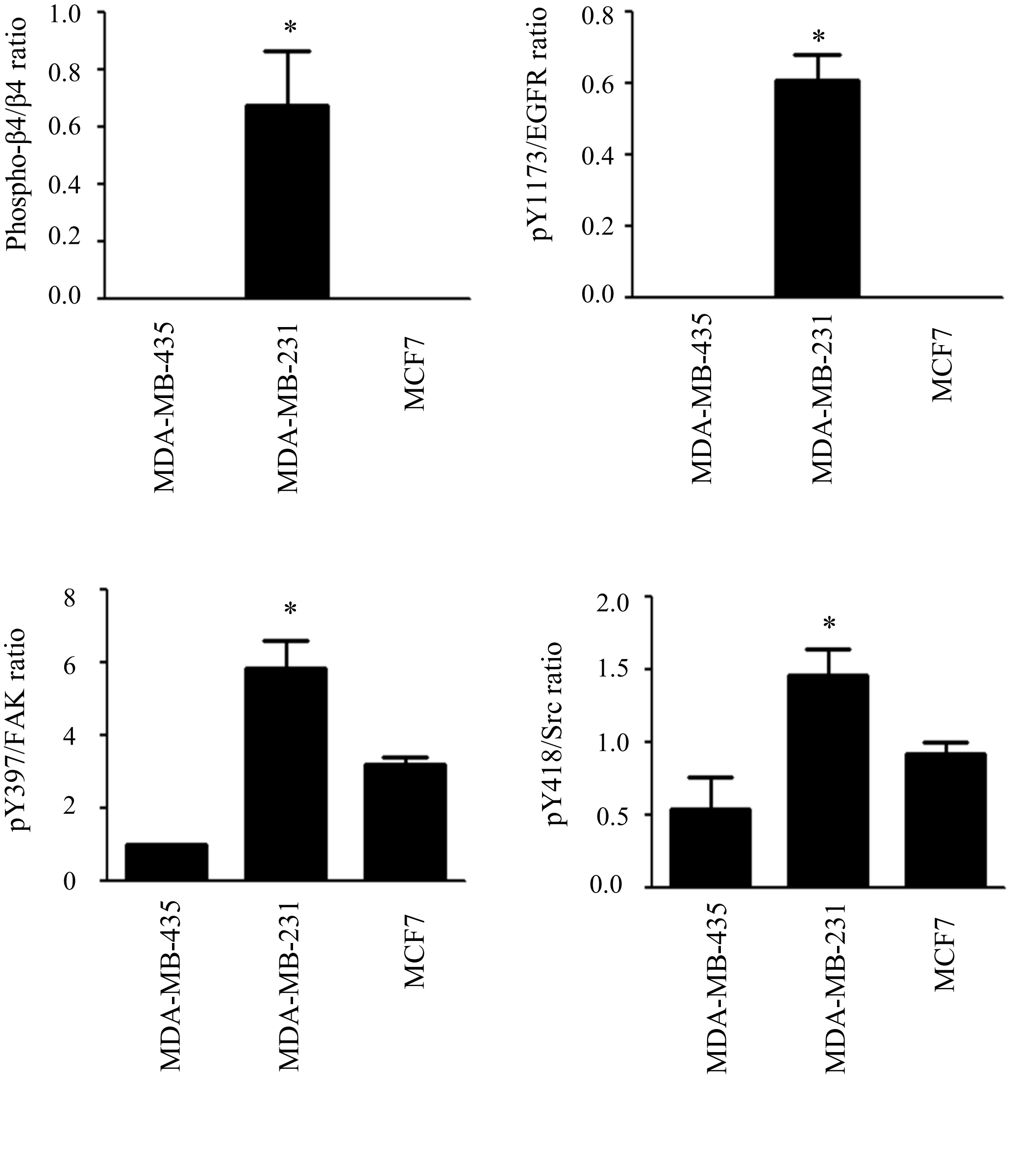

Supplementary Figure S6

Tai et. al.

Relative to Figure 2b

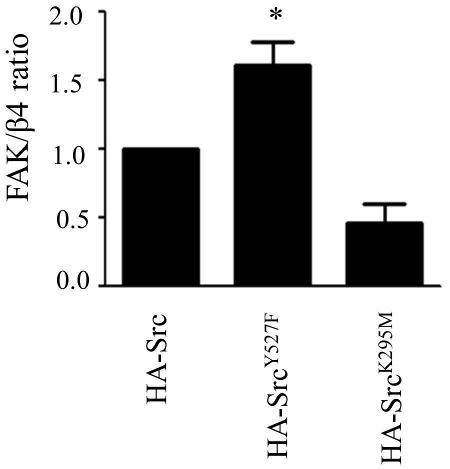

Relative to Figure 2c

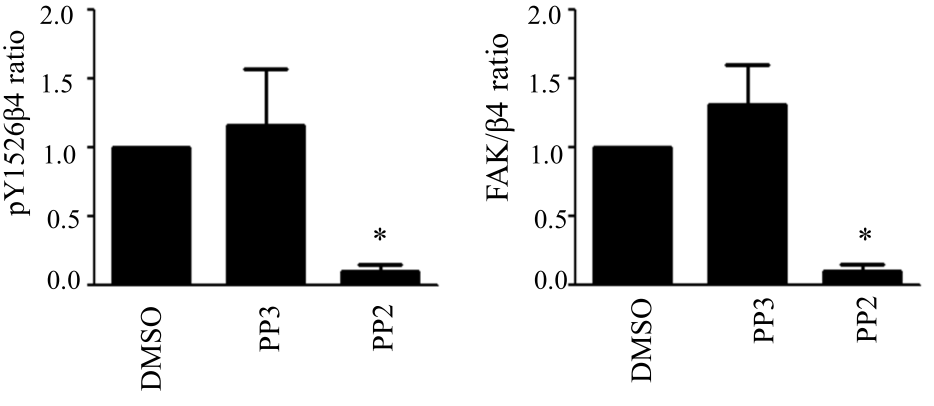

Relative to Figure 2d

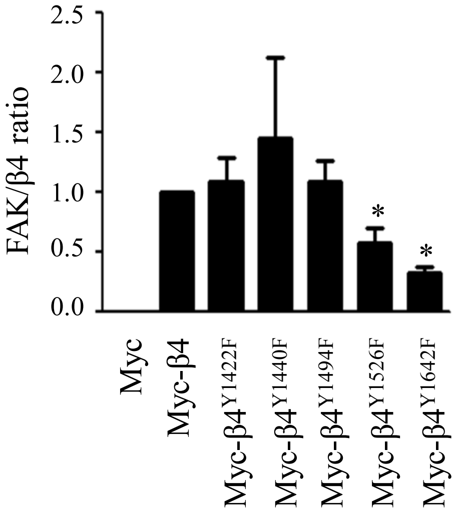

Relative to Figure 2e

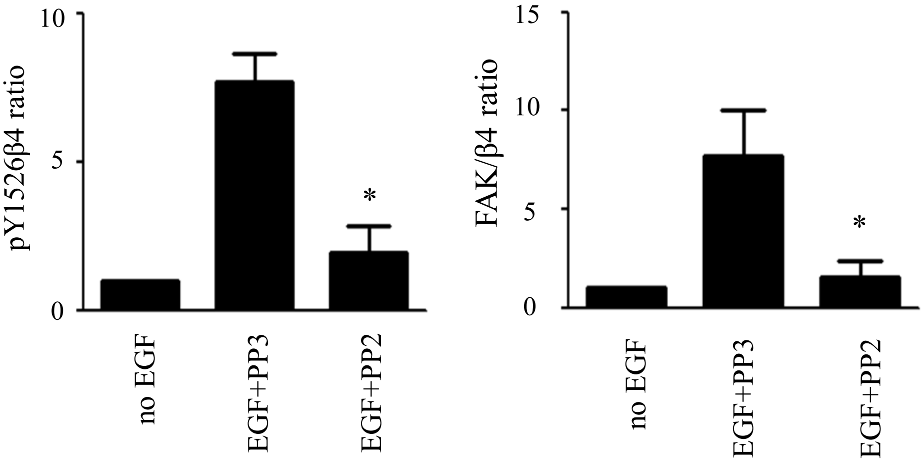

Supplementary Figure S6

Tai et. al.

Relative to Figure 3a

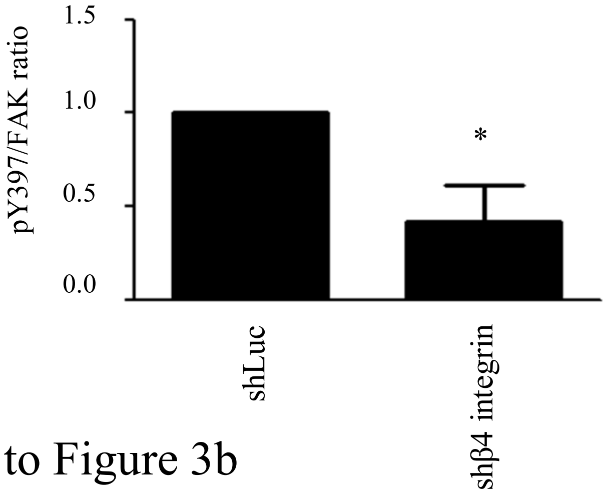

Relative to Figure 3b

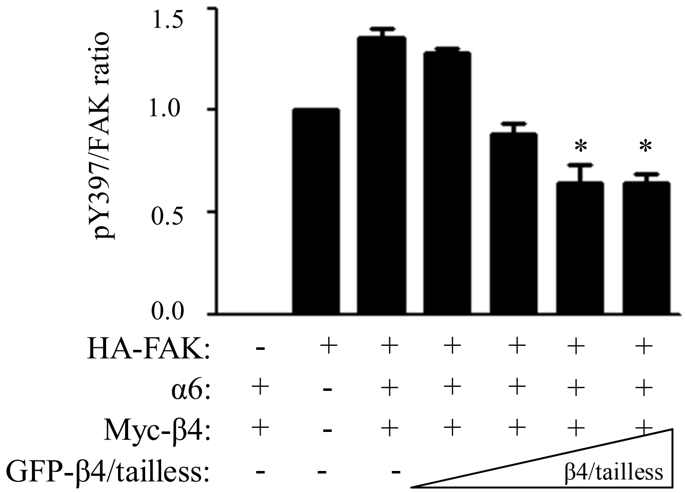

Relative to Figure 3c

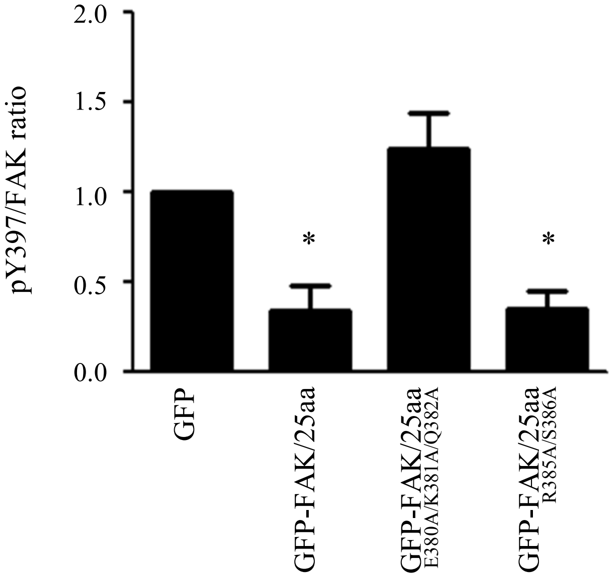

Supplementary Figure S6

Tai et. al.

Relative to Figure 3e

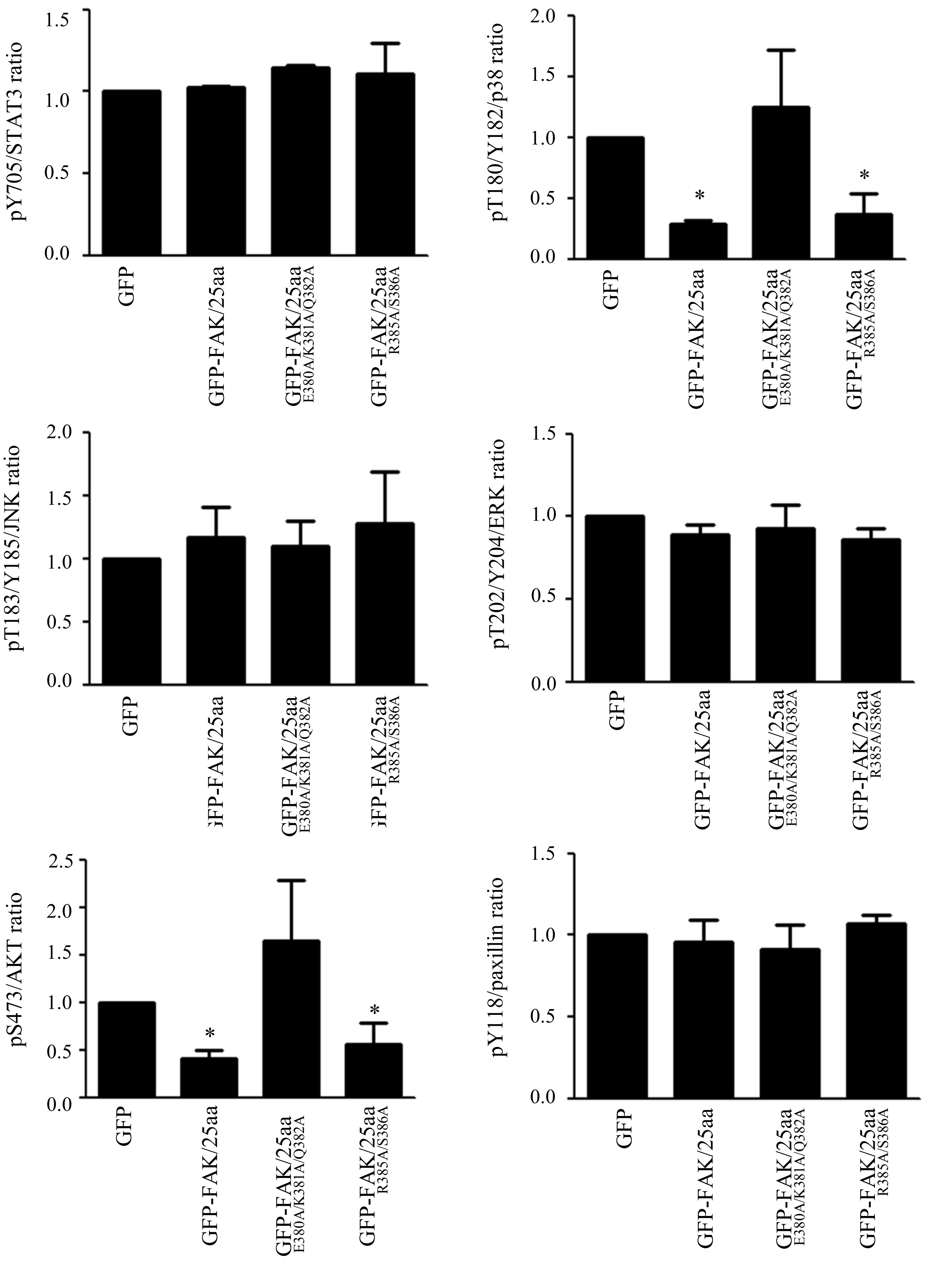

Supplementary Figure S6

Tai et. al.

Relative to Figure 4a

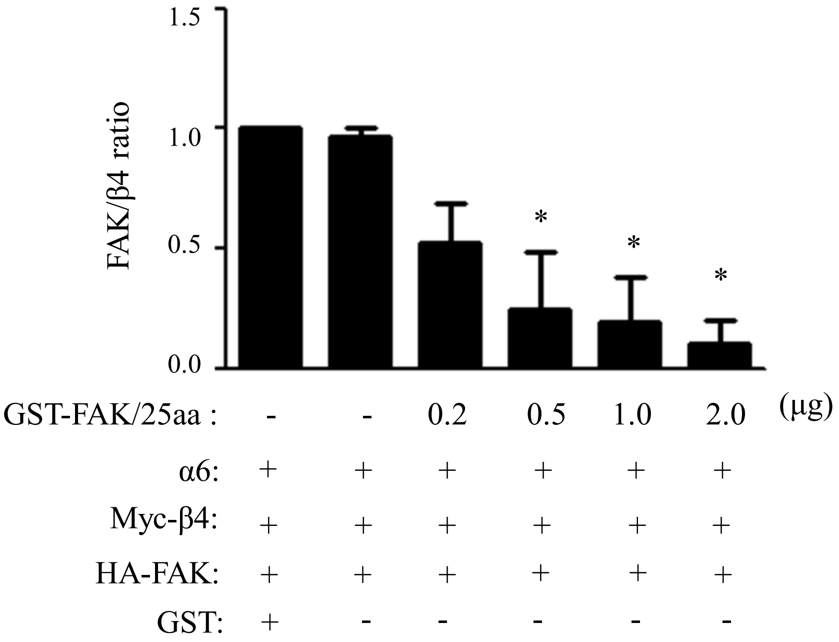

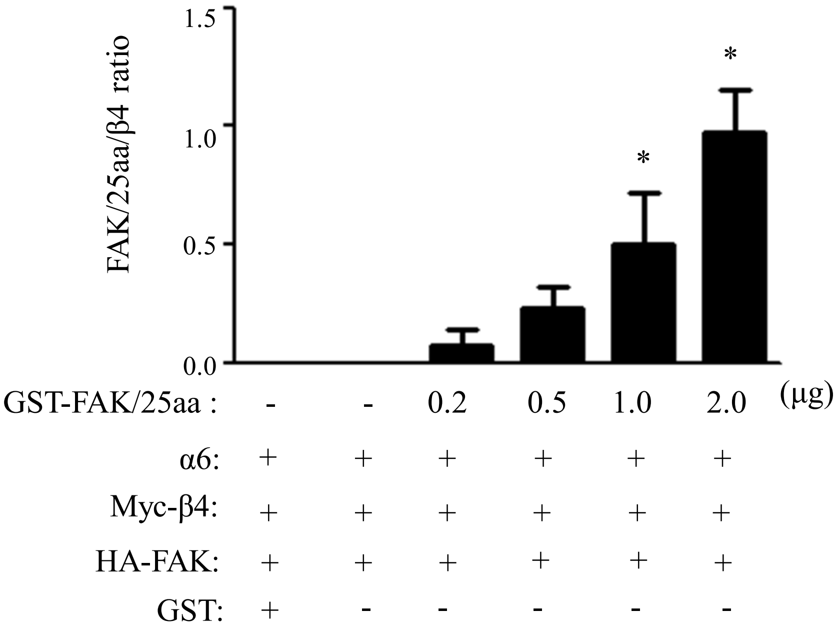

Supplementary Figure S7

Tai et. al.

Relative to Figure 1a

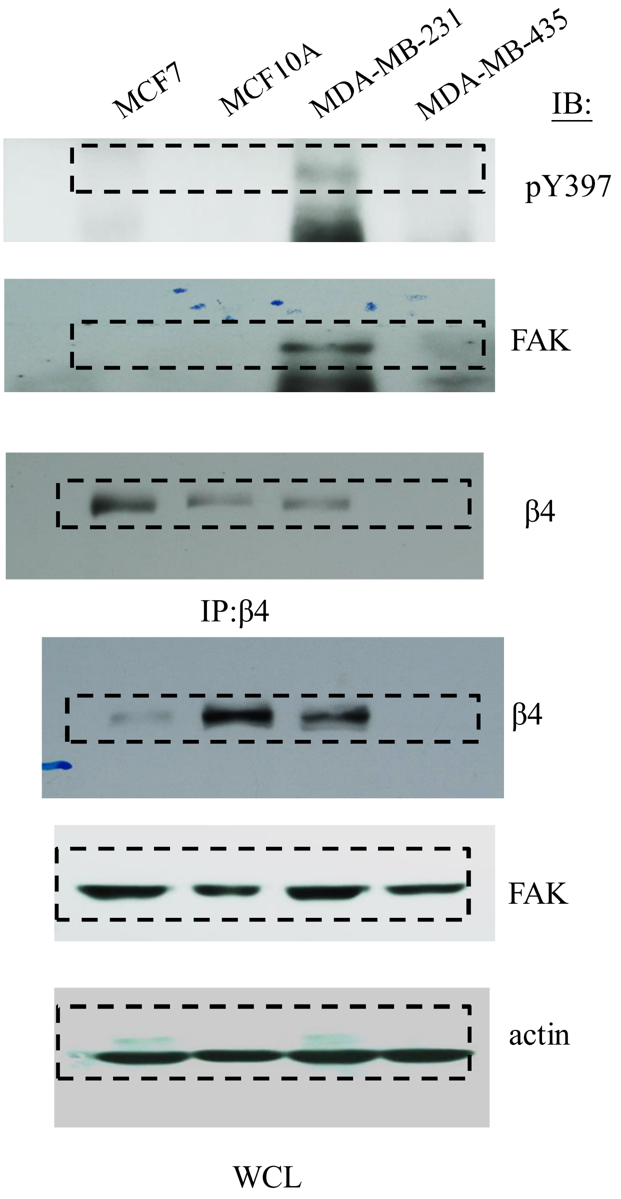

Relative to Figure 1c

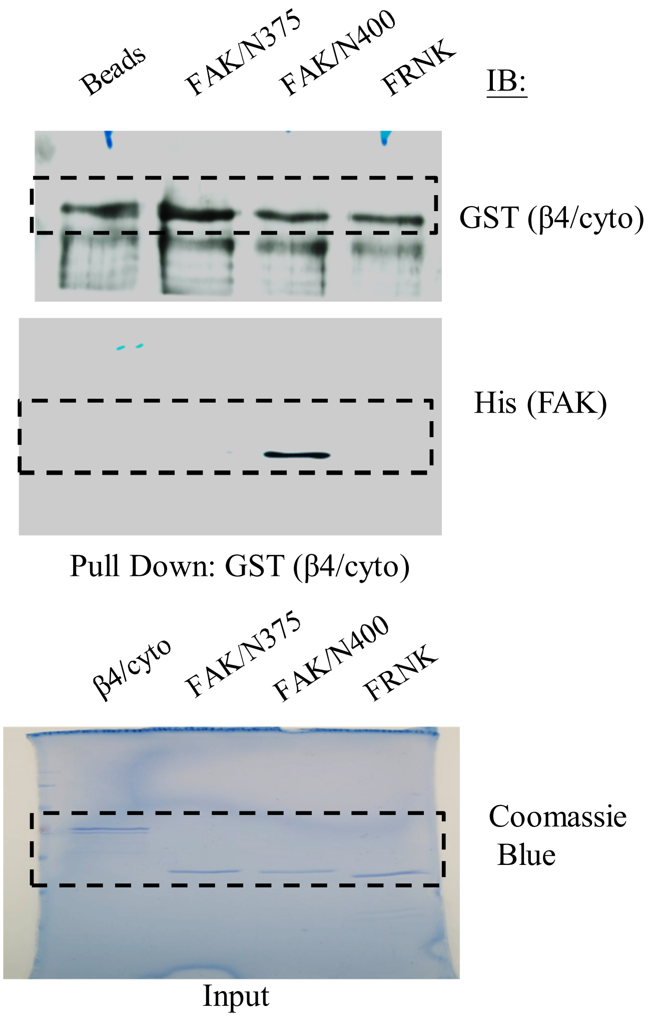

Supplementary Figure S7

Tai et. al.

Relative to Figure 1d

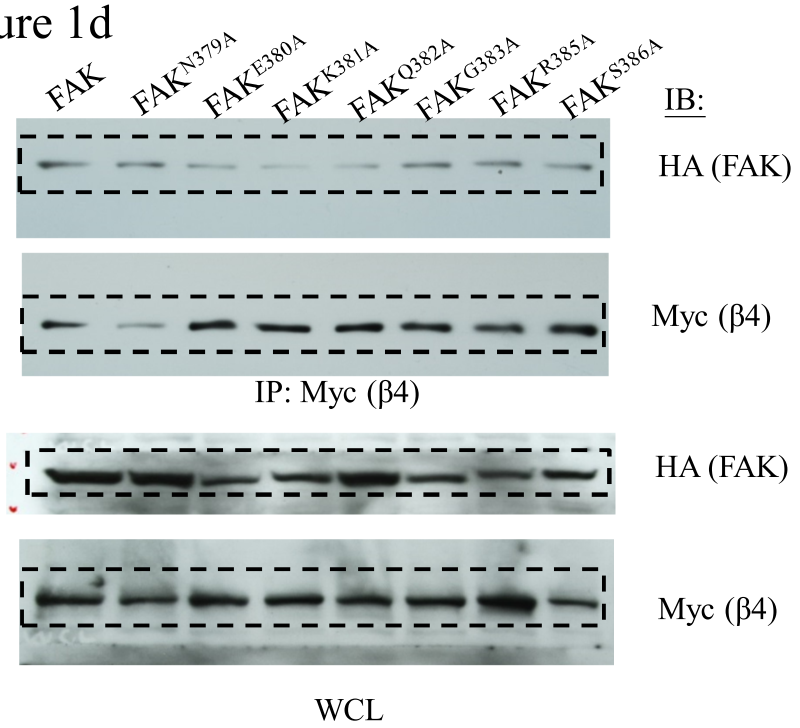

Relative to Figure 1e

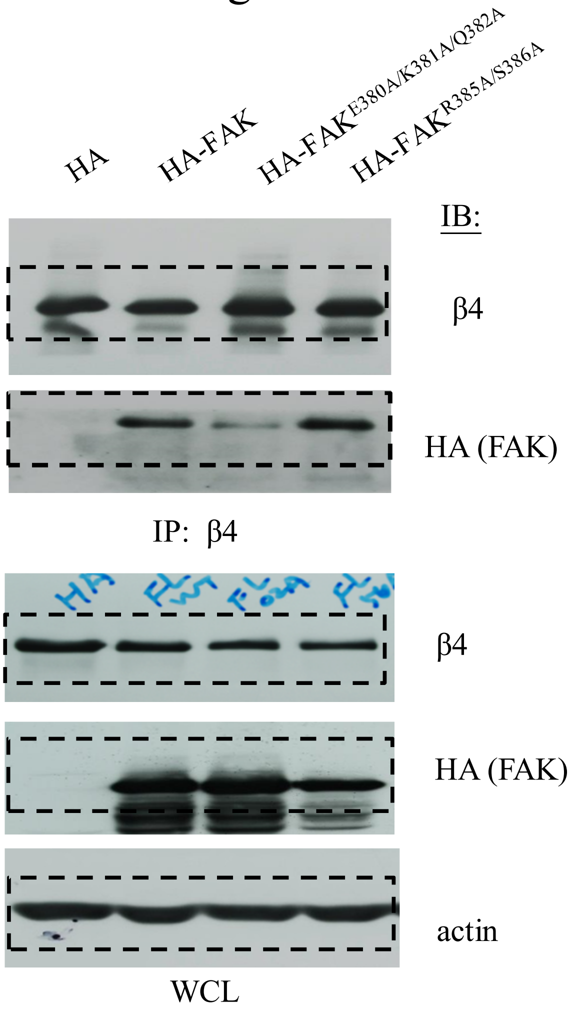

Relative to Figure 1f

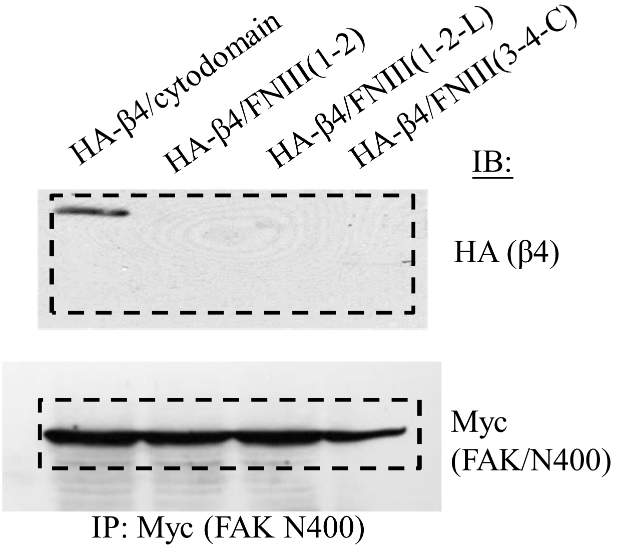

Supplementary Figure S7

Tai et. al.

Relative to Figure 2a

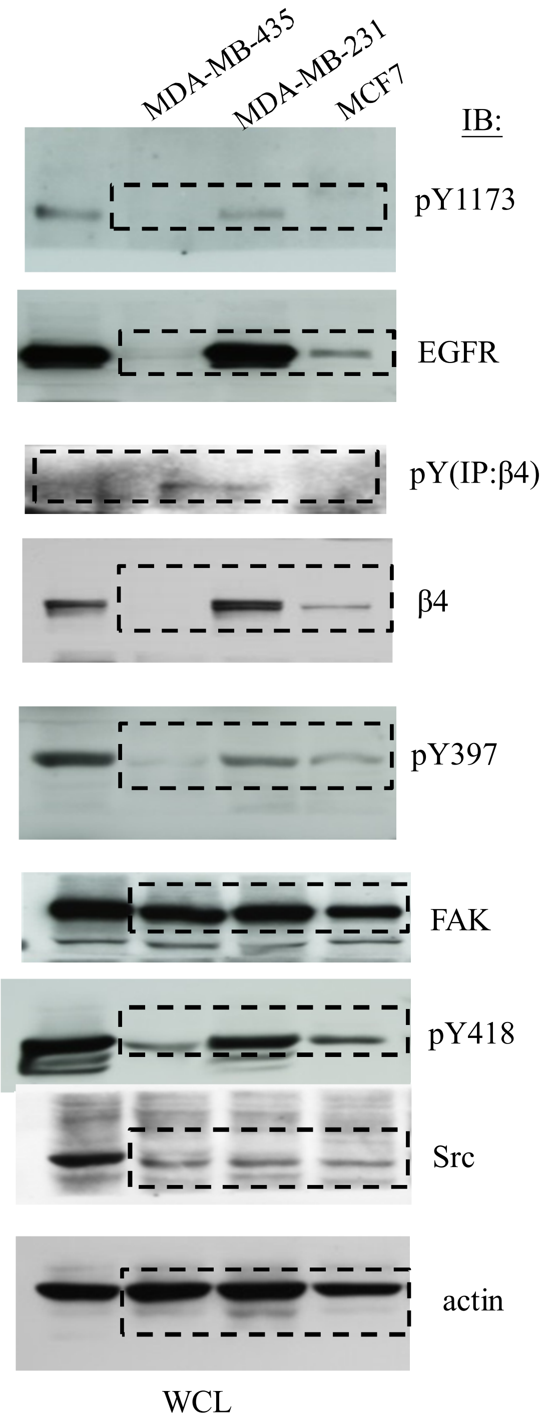

Relative to Figure 2b

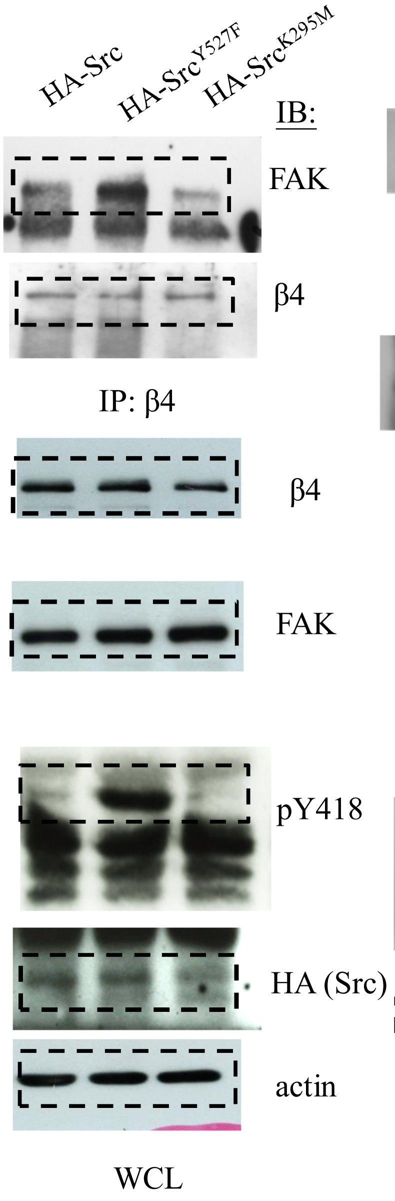

Relative to Figure 2c

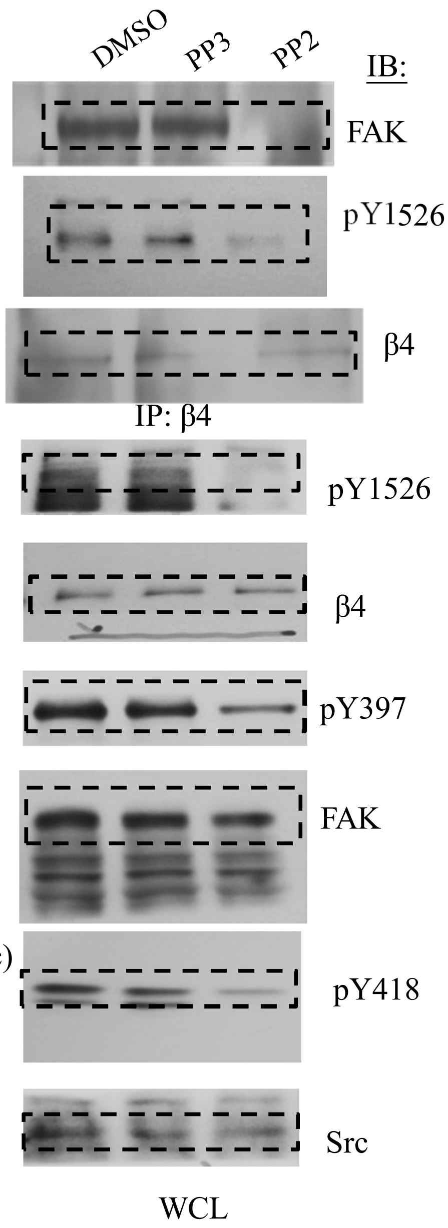

Supplementary Figure S7

Tai et. al.

Relative to Figure 2d

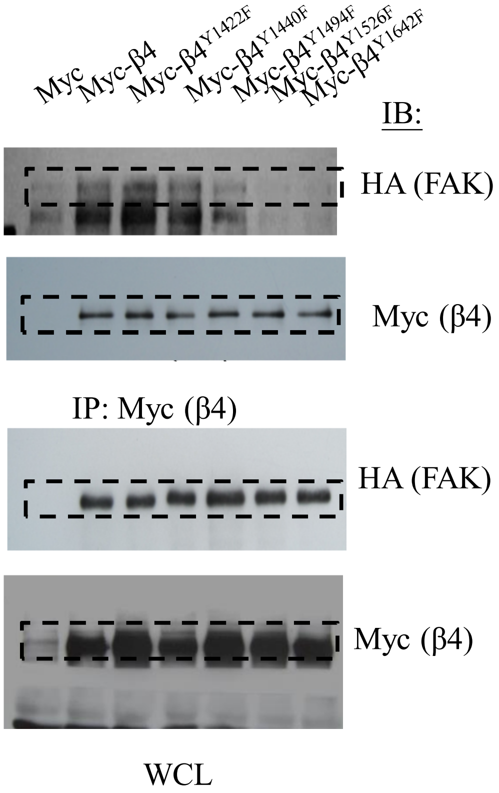

Relative to Figure 2e

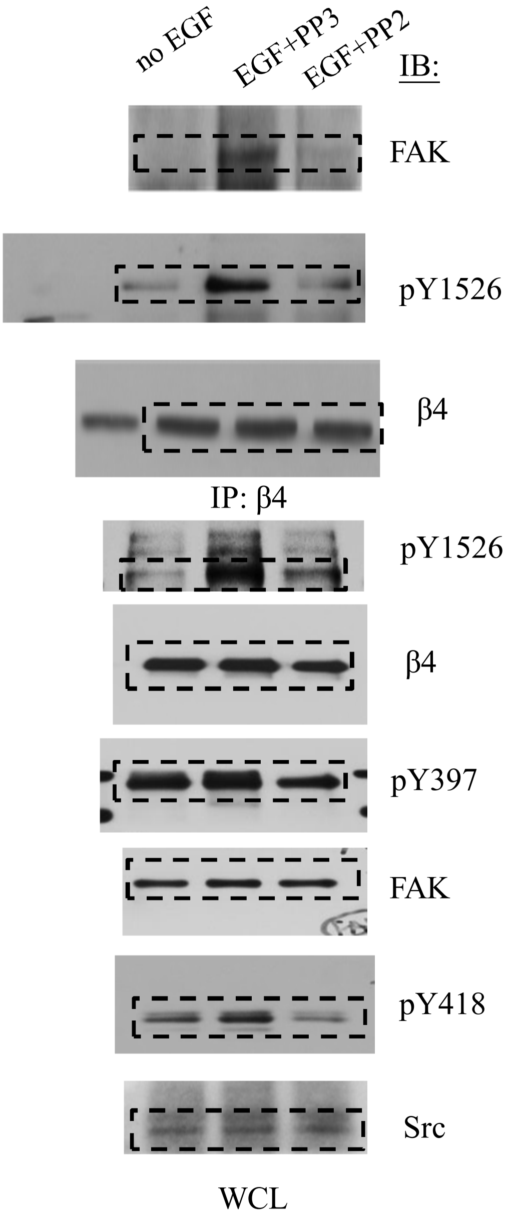

Supplementary Figure S7

Tai et. al.

Relative to Figure 3a

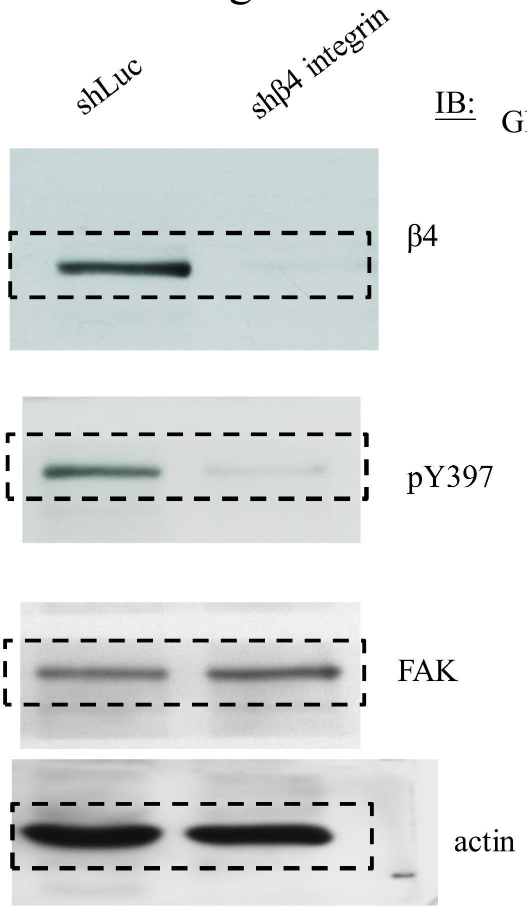

Relative to Figure 3b

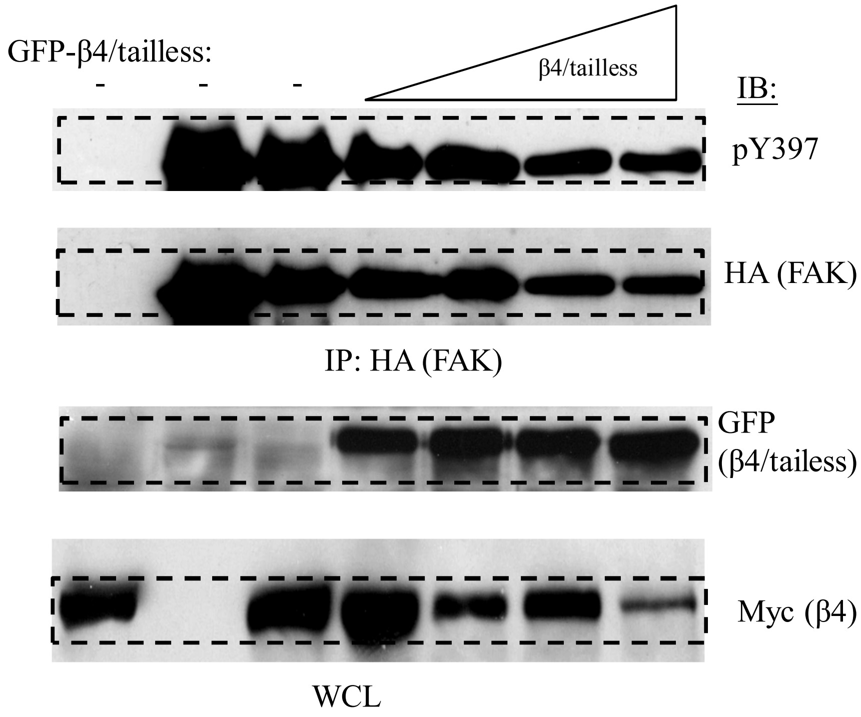

Relative to Figure 3c

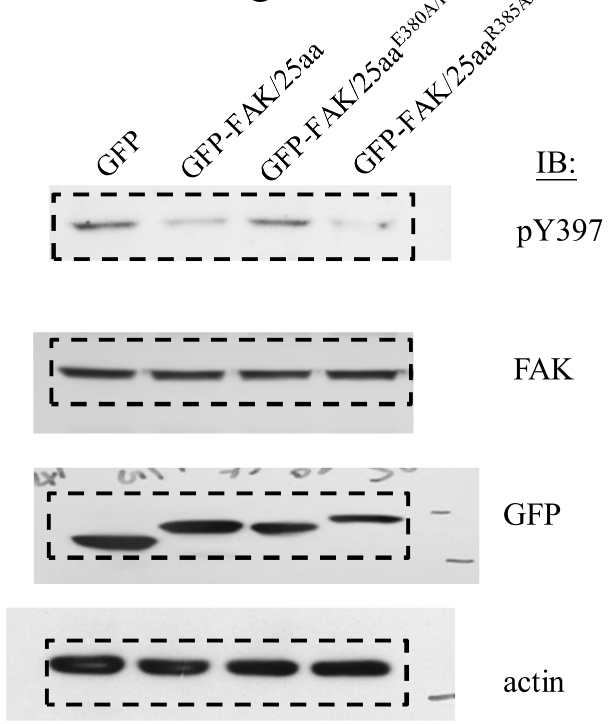

Relative to Figure 3d

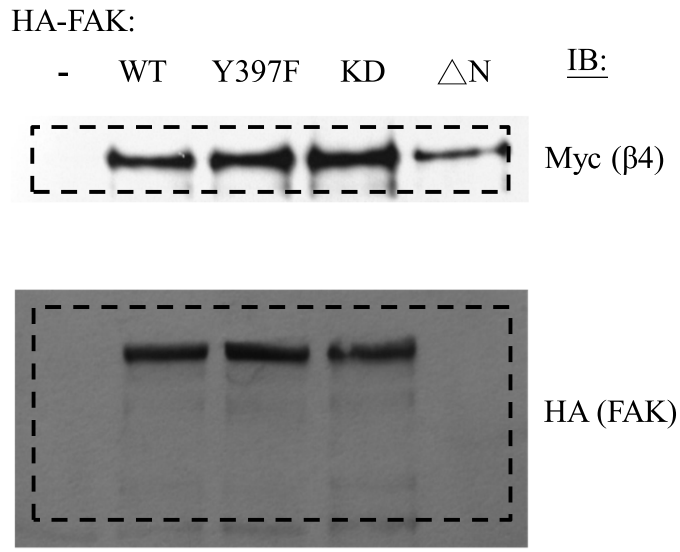

Supplementary Figure S7

Relative to Figure 3e

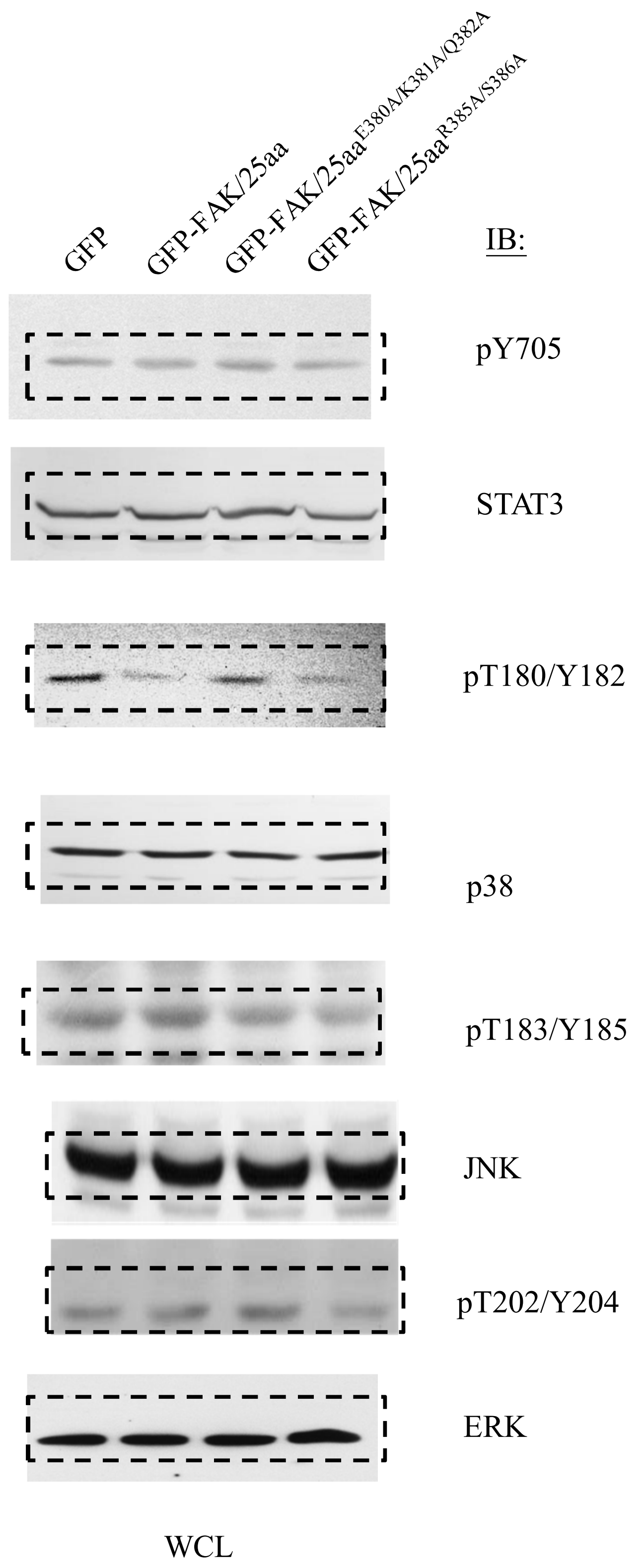

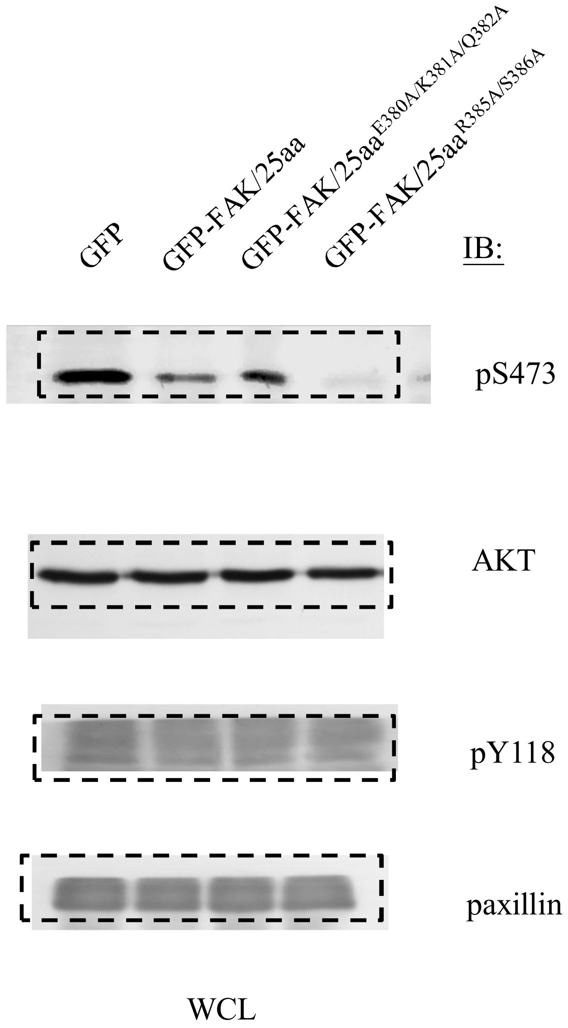

Supplementary Figure S7

Tai et. al.

Relative to Figure 4a

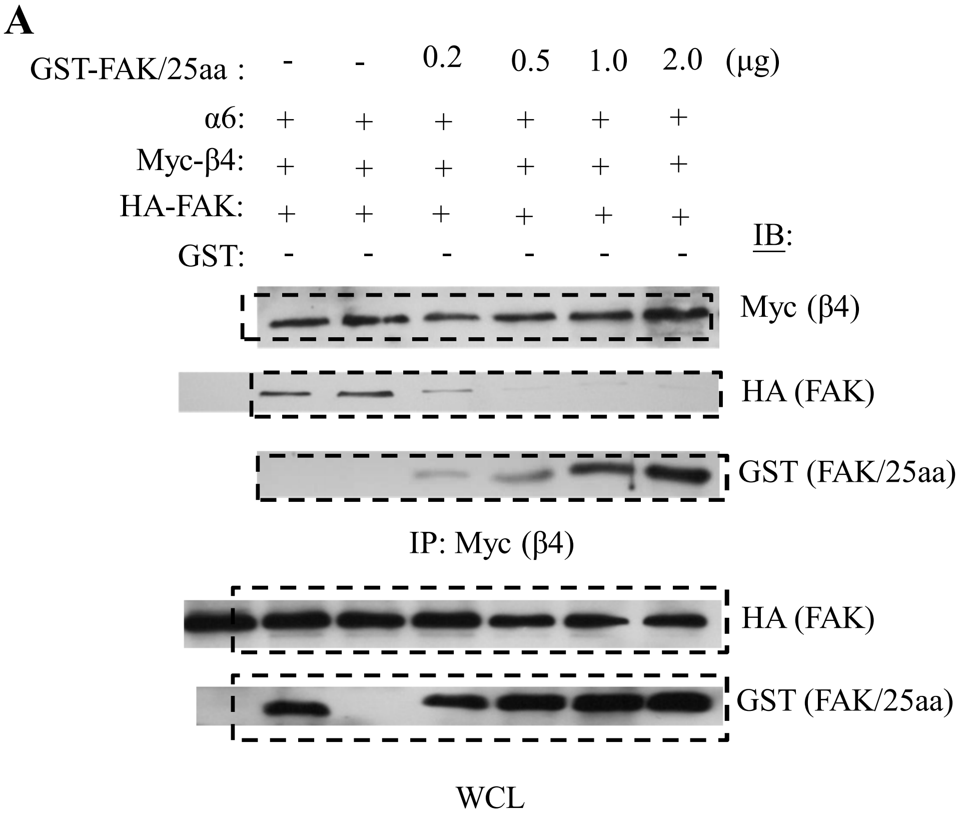

Relative to Figure 5a

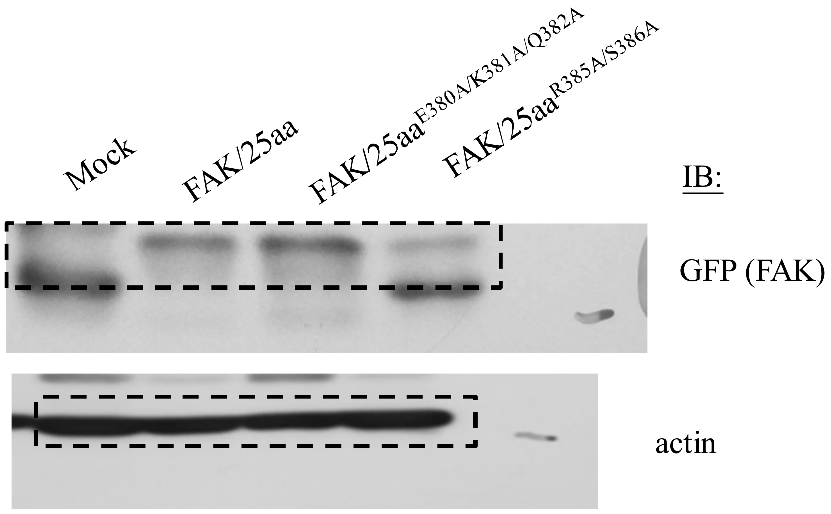

Supplementary Figure S7

Tai et. al.

Relative to Figure 6a

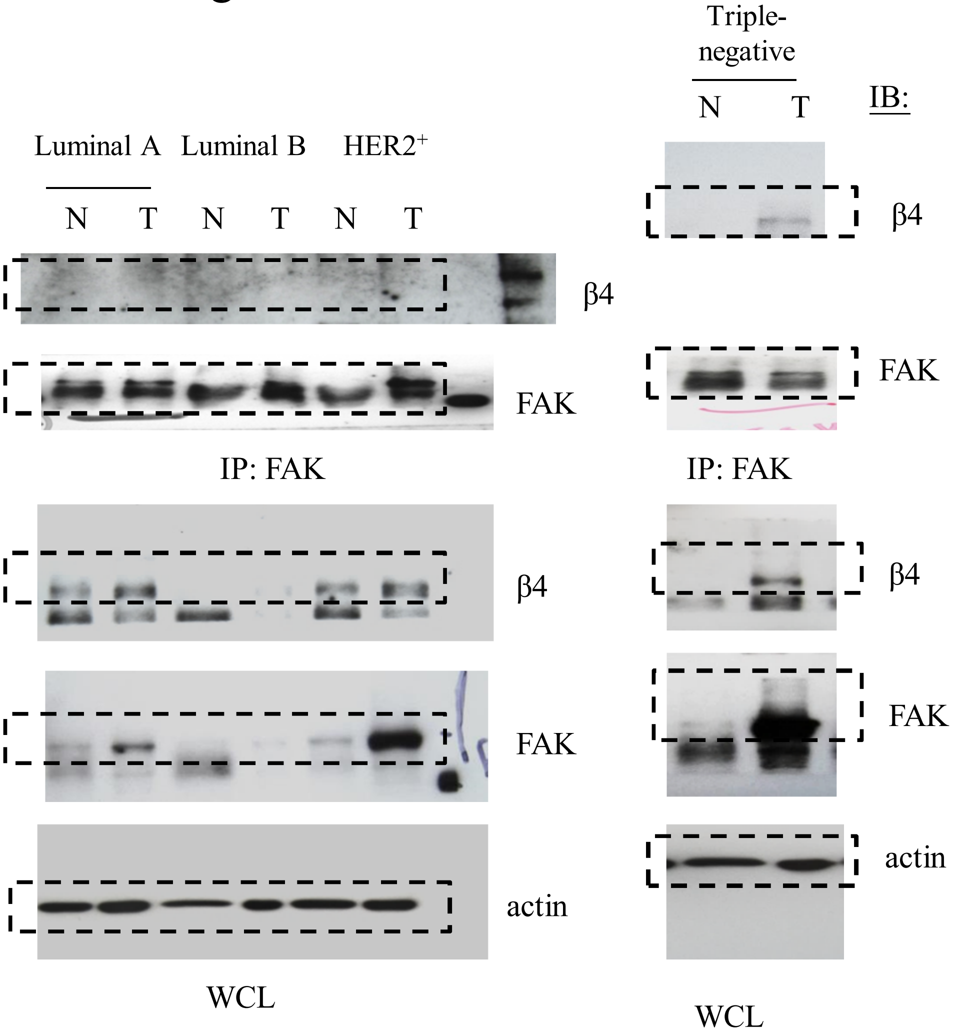

Supplement: Supplementary Information [file srep16408-s1.pdf]
